# Pulsatile MAPK Signaling Modulates p53 Activity to Control Cell Fate Decisions at the G2 Checkpoint for DNA Damage

**DOI:** 10.1016/j.celrep.2020.01.074

**Published:** 2020-02-18

**Authors:** Siddharth De, Callum Campbell, Ashok R. Venkitaraman, Alessandro Esposito

**Affiliations:** 1 Medical Research Council Cancer Unit, University of Cambridge, Hills Road, Cambridge CB2 0XZ, UK

**Keywords:** p53, MAPK/ERK pathway, DNA damage response, checkpoint signalling, signalling dynamics, cellular decisions, checkpoint fidelity

## Abstract

Cell-autonomous changes in p53 expression govern the duration and outcome of cell-cycle arrest at the G2 checkpoint for DNA damage. Here, we report that mitogen-activated protein kinase (MAPK) signaling integrates extracellular cues with p53 dynamics to determine cell fate at the G2 checkpoint. Optogenetic tools and quantitative cell biochemistry reveal transient oscillations in MAPK activity dependent on ataxia-telangiectasia-mutated kinase after DNA damage. MAPK inhibition alters p53 dynamics and p53-dependent gene expression after checkpoint enforcement, prolonging G2 arrest. In contrast, sustained MAPK signaling induces the phosphorylation of CDC25C, and consequently, the accumulation of pro-mitotic kinases, thereby relaxing checkpoint stringency and permitting cells to evade prolonged G2 arrest and senescence induction. We propose a model in which this MAPK-mediated mechanism integrates extracellular cues with cell-autonomous p53-mediated signals, to safeguard genomic integrity during tissue proliferation. Early steps in oncogene-driven carcinogenesis may imbalance this tumor-suppressive mechanism to trigger genome instability.

## Highlights

• DNA damage elicits opposing pro-survival and pro-arrest responses via MAPK and p53
• MAPK pulsations modulate p53-dependent transcription to determine cell fate
• MAPK/p53 signal dynamics control the stringency of the G2 DNA damage checkpoint
• MAPK/p53 integrate extracellular and intracellular cues to protect genome integrity

## Graphical Abstract

**Figure:**
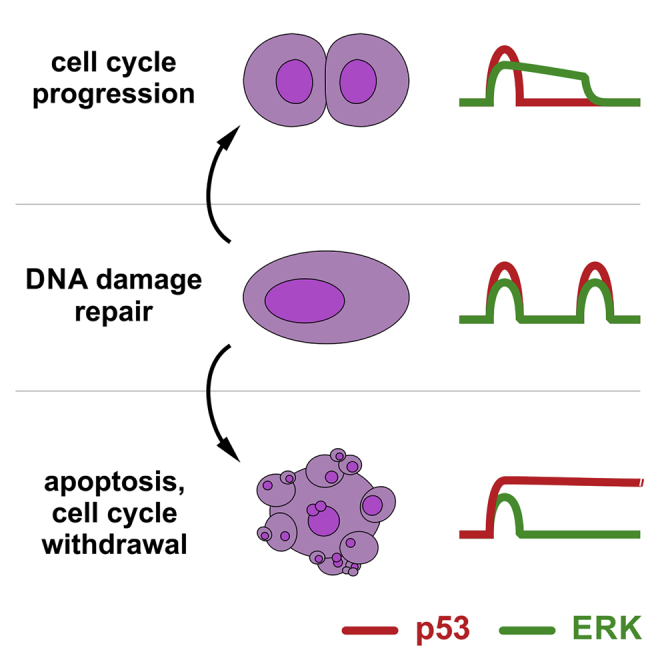

## Teaser

Cells coordinate repair and cell-cycle machinery in response to DNA damage to safeguard genomic integrity and tissue homeostasis. De et al. find that two opposing and pulsatile signals of pro-survival and pro-arrest networks permit cells to set a balance between distinct cell fates.

## Introduction

To maintain tissue function and homeostasis, mammalian cells respond to extracellular cues via signal transduction networks (STNs) that relay information from membrane receptors to the cell nucleus to influence cell fate decisions. The dynamic response of STNs (e.g., transient, oscillatory, or sustained) encodes fundamental information that controls cell fate (Purvis et al., 2012, Santos et al., 2007, Shankaran et al., 2009, Zwang et al., 2011), which is also recognized as a possible target for clinical modulation (Stewart-Ornstein and Lahav, 2017, Chen and Lahav, 2016).

Recent evidence suggests that cell fate decisions executed during processes that are seemingly cell autonomous may be regulated by STNs that respond to extracellular cues. One important example is the G2 cell-cycle checkpoint (Wei et al., 2010, Yan et al., 2007), which is activated by DNA lesions such as double-stranded DNA breaks (DSBs), and determines subsequent fates ranging from cell-cycle arrest to apoptosis (Shaltiel et al., 2015). Dynamic changes in the tumor suppressor p53 exemplify cell-autonomous mechanisms that control G2 checkpoint enforcement (Krenning et al., 2014, Batchelor et al., 2011, Loewer et al., 2010, Purvis et al., 2012). Pulses of p53 expression induced by DSBs mediate cell-cycle arrest, whereas sustained p53 activation instead triggers terminal cell fates, such as apoptosis or senescence (Batchelor et al., 2011, Purvis et al., 2012). These alternate fates are mediated through the differential transcriptional activation of downstream target genes that are dependent on the pattern (transient or periodic versus sustained) of upstream activators (Purvis et al., 2012, Loewer et al., 2010, Toettcher et al., 2009, Santos et al., 2007, Shankaran et al., 2009, Yamamoto et al., 2006). Emerging evidence (Yang et al., 2017) suggests that these processes may be influenced by extracellular cues such as growth-inducing signals, although the underlying mechanisms remain unclear. Such mechanisms are of central importance to understanding the complex response of multicellular tissues to DNA damage, which in turn underlies biological processes such as aging and carcinogenesis (Behrens et al., 2014, Ermolaeva et al., 2015). For instance, growing evidence implicates mitogen-activated protein kinase (MAPK) activation in the G2 checkpoint enforcement (Wang et al., 2007, Wei et al., 2010, Yan et al., 2007). MAPK signaling and its dynamics are known to control proliferation, influence the G1 DNA damage checkpoint (Santos et al., 2007, Zwang et al., 2011, Yamamoto et al., 2006, Tentner et al., 2012), and have a role at the G2 DNA damage checkpoint (Wei et al., 2010, Yan et al., 2007). However, it remains unclear mechanistically how MAPK dynamics exerts its influence, particularly in the G2 phase of the cell cycle and in the presence of DNA damage.

To address this issue, we have combined somatic cell genetics with optogenetics and single-cell biochemical imaging to investigate how the dynamic response of MAPK and p53 may intersect in determining cell fate during the G2 checkpoint. We reveal the previously unnoticed pulsatile activation of MAPK signaling after DNA damage and show how it intersects with the cell-autonomous control of p53 dynamics. We find that sustained ERK activation, typical of early steps during oncogene-driven carcinogenesis, enhances the phosphorylation of CDC25C, promoting the accumulation of pro-mitotic factors such as CYCLIN B1 and PLK1. Increased PLK1 activity in turn relaxes the stringency of the G2 checkpoint, permitting cells to evade prolonged or irreversible cell-cycle arrest post-DNA damage. Our findings provide fresh insights into how the DNA damage checkpoint, a seemingly cell-autonomous mechanism, integrates information from the environment and thus plausibly sets a balance between safeguarding genomic integrity and maintaining tissue homeostasis.

## Results

### MAPK Signaling Is Necessary for Recovery from the G2 Checkpoint

**Figure 1:**
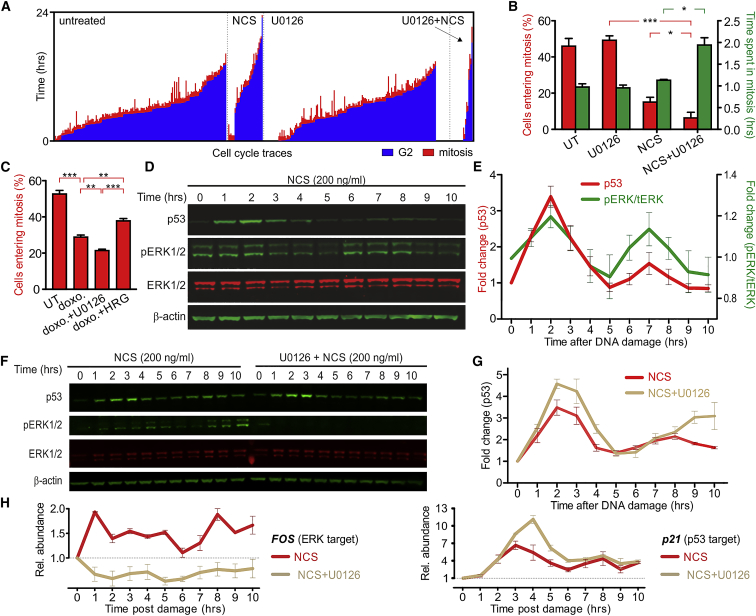
p53 and ERK Dynamics after DNA Damage Regulate Checkpoint Enforcement (A) Single-cell traces showing length of G2 (blue) and mitosis (red) for untreated and NCS-treated G2-synchronized MCF-7 cells, in the presence or absence of U0126 (n = 3). (B) Analysis of the single-cell traces shown in (A). When MAPK signaling is inhibited, the number of cells entering mitosis after DNA damage decreases significantly, and those cells entering mitosis spend a significantly longer time in mitosis. (C) Quantification of the number of synchronized MCF7 cells entering mitosis when treated with doxorubicin, along with U0126 and HRG. A larger number of cells entered mitosis when cells were treated with HRG in the presence of doxorubicin, while the number of cells entering mitosis when treated with U0126 was lower, indicating the enfeeblement of the G2 checkpoint (in the case of HRG) and higher stringency (in the case of U0126), upon the respective treatments (n = 3). (D) Representative western blot showing that the phosphorylation of ERK and p53 expression oscillate in phase after treatment of MCF-7 cells with NCS and effects of MEK inhibition on p53 expression (n = 3, means ± SEMs). (E) Quantification of p53 expression and fraction of phosphorylated ERK (n = 3). (F and G) Representative blot for p53 expression in response to DNA damage and relative stabilization in the presence of U0126 (F) and quantification (G) (n = 3). (H) Analysis of the expression (n = 3) of the pro-survival ERK target gene *FOS* and pro-arrest p53 target gene p21 in the presence of DNA damage and with the concomitant inhibition of MAPK signaling using U0126. See also Figures S1 and S2.

To investigate how MAPK exerts its influence over the DNA damage response, we used the human breast cancer cell line Michigan Cancer Foundation-7 (MCF-7), whose response to DNA damage has been extensively characterized (Batchelor et al., 2008, Batchelor et al., 2011). Initially, we tested whether MAPK signaling altered the G2 DNA damage checkpoint, as MAPK-dependent non-cell-autonomous mechanisms are poorly characterized in G2. Thus, we treated G2-synchronized MCF-7 cells (Figure S1A) with 200 ng/mL neocarzinostatin (NCS), a radiomimetic drug (Povirk, 1996, Smith et al., 1994) that primarily causes DSBs (Batchelor et al., 2011) with or without low concentrations of the well-characterized MEK inhibitor U0126 (Favata et al., 1998, Davies et al., 2002). We ensured careful sample processing to avoid MAPK activation by mechanical stimulation of the sample (see Figure S1B for a representative control and Method Details). At 1 μM, U0126 is specific for MEK (half-maximal inhibitory concentration [IC_50_] = 0.06 μM MEK1, 0.07 μM MEK2) and is sufficient to abrogate the MEK-mediated phosphorylation of ERK (Figure S1C). Live-cell imaging confirmed a DNA damage-induced arrest in G2, with only ∼15% of cells progressing to mitosis within the duration of the experiment, compared to 40% in the absence of NCS (Figures 1A and 1B). The inhibition of MAPK signaling in the presence of DNA damage further decreases the fraction of cycling cells (<10%), together with longer mitotic timing. In the absence of NCS, U0126 alone did not alter the fraction of cycling cells or length of mitosis, confirming that the observed changes in checkpoint stringency are specific for the G2 DNA damage checkpoint and not non-specific effects on the G2/M transition or cells that may be in a different cell-cycle phase. We also obtained similar results using doxorubicin (a topoisomerase 2 inhibitor that causes DNA damage; Figure 1C) and the near-diploid non-transformed immortalized human retinal pigment epithelium (hTert-RPE) cells (Figures S1D–S1F), thereby confirming that the MAPK response and its effect on G2/M progression is specific to neither MCF-7 cells nor NCS.

### DNA Damage Induces Oscillatory Activation of p53 and MAPK Signaling

To elucidate the mechanism of MAPK response, we quantified the MEK-dependent activating phosphorylation (pERK) of the extracellular signal-regulated kinases-1 and -2 (ERK) relative to total ERK (tERK), as surrogate measures of MAPK pathway activation. Irrespective of cell-cycle phase, ERK exhibits a peak of phosphorylation (pERK/tERK) at ∼2 h, followed by a second peak 5 to 6 h later (i.e., 7–8 h after NCS treatment; Figures 1D and 1E) after treatment with 200 ng/mL NCS. The activation of ERK exhibits a dynamic very similar to that already reported for the dampened oscillations in p53 expression after DNA damage (Batchelor et al., 2008, Batchelor et al., 2011, Loewer et al., 2010, Purvis et al., 2012). This coordinated response of MAPK with p53 has not been reported previously, and it is evident also in RPE-1 cells (Figures S2A and S2B).

### Damage-Induced MAPK Signaling Shapes p53-Dependent Transcriptional Programs

Mechanistically, p53 pulses maintain cells in an ambiguous state that enforces cell-cycle arrest and promotes DNA damage repair and cell survival by delaying cell death or senescence (Purvis et al., 2012). Therefore, we hypothesized that MAPK signaling may contribute to counteract p53-dependent mechanisms of cell-cycle arrest and withdrawal. While MEK inhibition alone has no effect on p53, in the presence of NCS-mediated DNA damage, U0126 further stabilizes p53, enhancing p53 expression in both MCF-7 and RPE-1 (Figures 1F, 1G, S2A, and S2B). The U0126-dependent stabilization may be caused by different levels of DNA damage or kinetics of repair in the presence or absence of U0126. Therefore, we measured the number of γH2AX foci per cell in MCF7 cells (a marker of DNA damage) by immunofluorescence at different times after exposure to NCS, again in the absence or presence of U0126 (Figures S1G and S1H). We observed no significant difference, suggesting that the stabilization of p53 observed is due to the regulation of the pathway by MAPK and not by the altered rate of DNA repair kinetics in the presence of the MAPK inhibitor.

Intermittent versus sustained activation of ERK (Aoki et al., 2013) or p53 (Purvis et al., 2012) upregulates the expression of distinct sets of genes, suggesting a possible MAPK-mediated mechanism of control of cell-cycle arrest. Thus, we analyzed the expression of transcripts encoding genes reported to be upregulated upon intermittent (downstream of ERK: *c-FOS*, *EGR1*, and *DUSP1*; downstream of p53: *p21*, *GADD45a*, and *XPC*) or sustained (ERK: *JUN-B* and *CTGF*; p53: *BAX*) ERK or p53 activation using quantitative real-time PCR analysis of messenger RNA (mRNA) up to 6 h after treatment with NCS (Figures S2D–S2G). Notably, the transient activation of ERK after DNA damage is sufficient to augment the transcript levels of genes reported to respond to pulsed activation such as *EGR1*, *c-FOS*, and *CTGF*, but it does not enhance to a detectable extent other targets such as *DUSP1* and *JUN-B* or most of these genes (*EGR1*, *CTGF*, *c-FOS*, and *JUN-B*) in the presence of U0126 (Figures S2E and S2F). Similarly, we observe that the expression of those transcripts sensitive to p53 pulses (e.g., *p2*1, *GADD45a*, *XPC*) are altered by the ablation of MAPK signaling, resulting in higher expression levels and altered kinetics (Figure S2G). At the same time, a gene such as *BAX*, which is insensitive to hour-long pulses of p53 (Purvis et al., 2012), remains unperturbed in the absence of MAPK signaling (Figure S2G). We also confirmed these observations on the transcriptional regulation of c-FOS and p21 with qPCR up to 10 h after DNA damage both in MCF-7 (Figure 1H) and RPE-1 (Figure S2C). These experiments thus demonstrate that a DNA damage-induced dynamic MAPK response does not solely alter p53 dynamics, but also significantly reshapes the dynamics and overall levels of both ERK and p53 downstream gene transcripts.

### p53-Independent and ATM-Dependent ERK Phosphorylation

The similarities between the dynamic behavior of p53 expression and ERK activation after DNA damage prompted us to hypothesize that p53, which forms a natural oscillator with its partner MDM2 (Lahav, 2008, Lahav et al., 2004, Stewart-Ornstein and Lahav, 2017), triggers MAPK signaling. However, small interfering RNA (siRNA)-mediated depletion of p53 neither abrogated the activation nor changed the dynamics of MAPK signaling after DNA damage (Figures S3A–S3C), thereby indicating that p53 pulses are not responsible for the activation of ERK, but instead, upstream DNA damage checkpoint mediators could be acting independently to trigger p53 and ERK activation.

DSBs trigger the pulsatile activation of p53 via the checkpoint kinase ataxia telangiectasia mutated (ATM) (Batchelor et al., 2011). Therefore, we tested whether ATM was also necessary to activate the MAPK pathway after DNA damage. When NCS-treated MCF-7 was treated with 10 μM Ku55933, a well-characterized ATM-specific inhibitor (Hickson et al., 2004, Stagni et al., 2015, Lau et al., 2005, Mandriota et al., 2010), downstream targets of ATM such as CHK2 and p53 were not phosphorylated, resulting in the absence of p53 pulses (Figures S3D–S3F). ATM inhibition also led to a reduction in the activation of ERK, thereby confirming that DNA damage is responsible for the transient activation of ERK in an ATM-dependent manner (Figure S3E).

### ERK-Dependent Effects Are Mediated by Stochastic Pulses of ERK Activity

**Figure 2:**
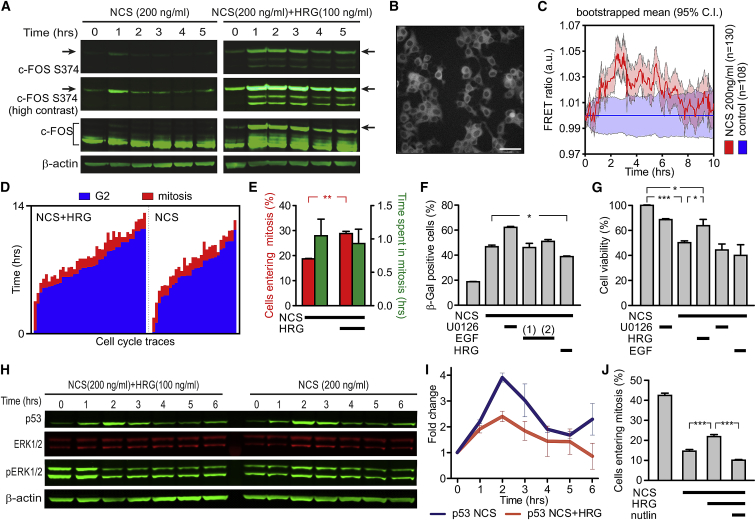
Transient or Sustained Activation of MAPK Signaling Differentially Controls the G2/M Checkpoint and Cell Fate after DNA Damage (A) c-FOS phosphorylation after DNA damage is transient, similar to the transient nature of EGF stimulation (Figure S4D) and different from the more stable response to HRG (n = 2). Arrows indicate c-FOS phosphorylated at Ser374. (B) Fluorescence images of the stable MCF7-EKAREV cell lines expressing an FRET-based ERK activity reporter. Scale bar: 50 μm. (C) Control-normalized bootstrapped averages (shaded areas represent the 95% confidence intervals) of ERK activity in response to DNA damage in MCF-7 cells from one representative experiment (n = 3). (D) Single-cell traces of MCF-7 cells treated with NCS in the presence or absence of HRG (n = 3) showing cells recovering from G2 arrest in the presence of HRG treatment. (E) Analysis of the single-cell traces showing that HRG does not alter the length of mitosis contrary to U0126 but releases cells into mitosis. (F) β-Galactosidase staining of control and NCS-treated MCF7 cells in the presence of the MAPK inhibitor U0126, a single or multiple pulses of EGF and HRG, 4 days after treatment. Quantification of the β-galactosidase staining showing the increased survival of cells treated with HRG in the presence of NCS as compared to NCS-treated cells alone (n = 3). (G) Cell viability of NCS-treated RPE-1 cells in the presence of U1026, single pulse of EGF and HRG, 3 days after treatment. Quantification of cell viability shows the increased survival of cells treated with HRG in the presence of NCS as compared to NCS-treated-only cells (n = 3). (H) Representative western blot showing that p53 expression after DNA damage is reduced by HRG in asynchronous cells (n = 2). (I) Quantification of the data shown in (H). (J) Quantification of the number of cells entering mitosis when treated with Nutlin in the presence of NCS, and HRG shows the rescue of G2 arrest (n = 3). Data shown as means ± SEMs. See also Figures S3 and S4.

To verify that the DNA damage-dependent oscillatory phosphorylation of ERK alters phenotypic outcomes in addition to its effect on p53 expression, we analyzed the phosphorylation of c-FOS at serine 374 (S374). Typically, c-FOS S374 is transiently phosphorylated by ERK, resulting in a partial stabilization that can be strengthened only when c-FOS is also phosphorylated at S324 upon sustained ERK activity (Murphy et al., 2002). We observed c-FOS stabilization and phosphorylation at S374 within the first hour after DNA damage, followed by a fast decrease to basal levels (Figure 2A). This result confirms that ERK pulsatile activation exhibits tangible effects at the transcriptional and post-translational levels that are known to be specific to transient (e.g., similar to an epidermal growth factor [EGF] response; Figures S4B and S4D) rather than sustained ERK activity (Aoki et al., 2013) (e.g., similar to a heregulin [HRG] response; Figure S4C).

At single-cell resolution, ERK kinase activity either exhibits stochastic pulses in the absence of external stimuli (Aoki et al., 2013) or is activated in discrete pulses upon activation by growth factors such as EGF (Santos et al., 2007, Shankaran et al., 2009). To determine the nature of ERK activation at the single-cell level after DNA damage, we generated an MCF-7 cell line stably co-expressing the fluorescence resonance energy transfer (FRET)-based EKAREV biosensor and H2B-mCherry (MCF7-EKAREV; Figures 2B and S3G). This cell line reports on the ERK-dependent phosphorylation of the CDC25C-derived peptide around threonine 48. We validated this reporter cell line by monitoring responses to well-characterized mitogenic growth factors EGF (130 ng/mL) and HRG (100 ng/mL). As expected, EGF and HRG resulted in well-defined transient and sustained responses, respectively, both at a single-cell level (Figures S3H and S3I) and as population measurements (Figures S4A, S4C, and S4D). On the contrary, upon treatment with NCS, MCF7-EKAREV cells exhibited a prototypical stochastic activity of ERK (Aoki et al., 2013), which must be averaged over a population of cells (Figure 2C; n = 130 for NCS and n = 108 for control) to recapitulate observations on ERK phosphorylation from population ensembles. Thus, the phosphorylation of ERK and its subsequent increased activity, although seemingly stochastic at the single-cell level, results in a net increase in the phosphorylation of substrates such as c-FOS (Figure 2A), thereby speaking to the relevance of the pulsatile activation of the MAPK pathway post-DNA damage on its downstream targets.

### ERK Temporal Dynamics Dictates G2 Checkpoint Enforcement

To provide further evidence supporting this notion, we modulated the temporal dynamics of ERK activity during G2 arrest by using EGF and HRG as tools. EGF and HRG are growth factors known to trigger transient (minutes long) or sustained (hours long) activation of ERK above basal levels, respectively (Cohen-Saidon et al., 2009, Nakakuki et al., 2010). Thus, we treated MCF-7 cells with either EGF at 130 ng/mL or HRG at 100 ng/mL, concentrations that were selected to obtain similar peak responses of ERK activity as measured by ERK phosphorylation in western blots (Figures S4C and S4D). EGF-induced transient ERK activation did not change the fraction of cells entering mitosis after the exposure of G2-synchronized cells to NCS (Figure S4E). In addition, transient stimulation with EGF at subsequent time points did not alter the timing or fidelity of the G2 DNA damage checkpoint (Figure S4F).

However, when G2-arrested MCF-7 cells were treated with HRG and NCS, we observed a 50% increase in cycling cells compared to NCS-treated cells alone (Figures 2D and 2E). Similar results were also obtained when cells were treated with doxorubicin (Figure 1C). We did not detect significant changes in the length of G2 in NCS- or HRG-treated cells (Figure S4H), indicating that a sustained mitogenic signal does not shorten the length of G2 arrest, but instead increases the likelihood that cells transit into mitosis. MCF-7 cells are defective in apoptosis (Purvis et al., 2012), but they can trigger senescence in response to DNA damage. Therefore, we scored MCF-7 cells for senescence 4 days after treatments with NCS to test the long-term survival of cells after the manipulation of MAPK dynamics in G2-arrested cells. We observed an increased number of senescent cells when cells in the presence of DNA damage were treated with U0126, no changes for EGF pulses, and significantly fewer senescent cells with HRG with respect to the cells treated only with NCS (Figures 2F and S4I). We also tested whether modulation of MAPK signaling during the DNA damage response would result in differential survival in RPE-1 cells. RPE-1 cells were treated similarly to MCF-7 cells, but as RPE-1 cells can trigger apoptosis, we assessed their viability by CellTiter-Blue 3 days after treatment with NCS. Damaged cells showed higher viability when treated with HRG compared to cells treated with either EGF, U0126, or NCS alone (Figure 2G), confirming the importance of MAPK signaling dynamics in the determination of cell fate during the DNA damage response.

Furthermore, we confirmed that HRG but not EGF decreases p53 expression (Figures 2H, 2I, and S5A–S5D) through a similar mechanism involving the MDM2-MDMX axis (Gerarduzzi et al., 2016; data not shown), again suggesting that MAPK dynamics is crucial to establish a balance between cell-cycle arrest and progression in a p53-dependent manner. The p53 levels could be rescued by treating the cells with U0126 in the presence of HRG and DNA damage (Figures S5E and S5F). Aiming to further test whether the dynamic cooperation between p53 and MAPK signaling contributes to the determination of G2/M progression during the DNA damage response, we attempted to rescue the stringency of the G2/M checkpoint by treating cells with Nutlin in the presence of HRG. Nutlin is a well-characterized MDM2 inhibitor (Purvis et al., 2012, Porter et al., 2016) that causes the stabilization of p53. To achieve stable expression of p53 during DNA damage, we used a Nutlin treatment strategy similar to the one established previously by Purvis et al. (2012). Figure 2J shows that Nutlin can rescue the G2/M checkpoint arrest in the presence of DNA damage and HRG (see also Figure S4J), confirming our previous conclusions. Hence, taken together, our results provide multiple lines of evidence for a model wherein dynamic changes in MAPK signaling regulate the cell fate decision between cell death or sustained G2 arrest versus transition into mitosis.

### Optogenetic Validation of MAPK-Dependent Cell Fate Decisions at the G2 Checkpoint

**Figure 3:**
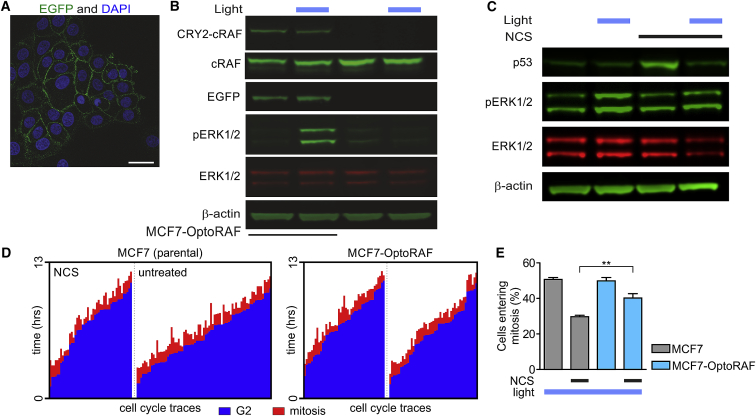
Sustained MAPK Signaling Attenuate p53 Response and Cell-Cycle Arrest (A) Confocal image of MCF-7 stably expressing the EGFP-CIBN plasma membrane anchor of the OptoRaf system. Scale bar: 50 μm. (B) Western blot analysis confirms the expression of both EGFP-CIBN and the cargo CRY2-cRAF (upper band in cRAF blot) and the light-inducible activation of MAPK signaling as measured by the phosphorylation of ERK after a 20-min exposure to blue light. (C) Representative western blot analysis confirms the reduction of p53 expression levels in the presence of sustained light activation with consequent sustained activation of the MAPK pathway (n = 2). (D) Single-cell traces of G2-synchronized control or NCS-treated MCF-7 and MCF7-OptoRaf cells showing the length of G2 (blue) and mitosis (red) in the presence of constant blue light stimulation. (E) Analysis of single-cell traces confirming that sustained activation of MAPK signaling increases the number of cells entering mitosis after G2 arrest (n = 3, means ± SEMs).

To directly determine whether MAPK activity regulates cell fate decisions at the G2 checkpoint, we engineered a stable cell line (MCF7-OptoRaf; Figure 3A) expressing a light-activatable C-RAF (Aoki et al., 2013) protein, the apical MAPK, capable of inducing specific ERK activation. After exposure to blue light, OptoRaf translocates to the plasma membrane, where it can interact and activate MEK and, consequently, ERK. After exposure to 20 min of blue light, MCF7-OptoRaf cells exhibit increased phosphorylation of ERK compared to both unlit MCF7-OptoRaf and (lit or unlit) MCF-7 cells. MCF7-OptoRaf cells thus permit us to activate MAPK signaling selectively (Figure 3B). Optogenetics-mediated sustained activation of MAPK signaling also results in a net decrease of p53 levels, similar to that observed by pharmacological intervention with HRG (Figure 3C). Sustained activation of RAF during live cell imaging resulted in a significant increase in cells progressing into mitosis in the presence of DNA damage compared to parental cells exposed to equal light intensities (Figures 3D and 3E). Therefore, this result confirms that sustained ERK activation is necessary and sufficient to alter G2/M checkpoint recovery and suggests that it acts independently of other pathways. These results provide a first demonstration that cell fate decisions following the G2 checkpoint for DNA profoundly depend on MAPK signaling and its dynamics.

### Sustained MAPK Activity Promotes Mitotic Entry despite the Presence of DNA Damage

**Figure 4:**
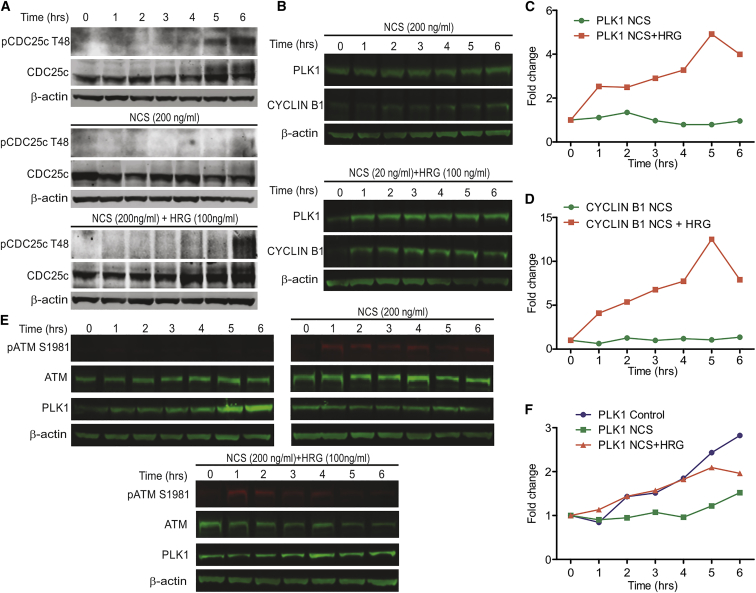
Sustained MAPK Signaling Allows Cells to Enter Mitosis after Damage-Induced G2 Arrest (A) Representative western blot showing that CDC25C phosphorylation at T48 in synchronized MCF-7 cells is decreased in response to DNA damage and is restored by HRG. (B) Representative western blot showing the expression levels of PLK1 and CYCLIN B1 in asynchronous MCF-7 cells after DNA damage, in the absence or presence of HRG. (C) Quantification of the western blot showing an HRG-dependent increase in PLK1, even in the presence of NCS. (D) Quantification of the western blot showing an HRG-dependent increase in CYCLIN B1, even in the presence of NCS. (E) Representative western blot showing the expression of PLK1 in G2-synchronized MCF-7 cells in control and NCS-treated cells and in NCS- and HRG-treated cells. (F) Quantification of the western blot showing that HRG can restore PLK1 expressing in synchronous cells to levels similar to undamaged cells. All of the experiments represent at least 3 independent repeats. See also Figures S5 and S6.

**Figure 5:**
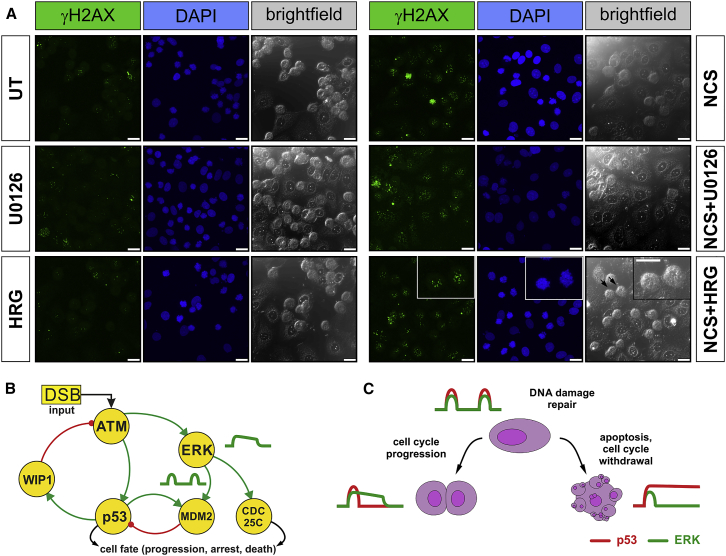
Cells Enter Mitosis with Unresolved DNA Damage (A) Cells stained against phosphorylated H2AX, a marker of DNA damage visible in any phase of the cell cycle, showing the presence of unresolved DNA damage in cells that have entered mitosis (marked by arrows). Accompanying bright-field images clearly show rounded mitotic cells in the field of view. There is little or no damage in mitotic cells, which have either been left untreated or treated with U0126 or HRG alone. Scale bar: 25 μm. (B) Graphical representation of the positive and negative feedback first identified by Lahav et al. (2004), subsequently modeled with the addition of the WIP1 phosphatase role in Batchelor et al. (2011), where ERK activity discovered in this work is exemplified with the reported MAPK-MDM2 interaction (Gerarduzzi et al., 2016). (C) A simple competitive model for the DNA damage checkpoint. The pulsatile response of p53 is known to maintain DNA-damaged cells in an arrested state conducive to DNA damage repair without permanent withdrawal from the cell cycle. The pulsatile activation of the MAPK/ERK pathway after DNA damage is necessary for the maintenance of this pro-survival state. The loss of MAPK/ERK activation leads to increased p53 activity and an increased probability of permanent cell-cycle arrest, while sustained MAPK/ERK activation leads to a greater probability of checkpoint recovery from DNA damage-induced arrest mediated by CDC25C phosphorylation.

Since sustained MAPK activation suffices to decrease p53 levels (and rescued in the presence of MAPK inhibitor U0126) and increases the frequency of cells progressing into mitosis after DNA damage (Figures 3 and S5C–S5F), we hypothesized that sustained ERK activation via HRG, but not transient activation via EGF, may increase the expression of pro-mitotic proteins (e.g., CYCLIN B1, PLK1) and the ERK-dependent phosphorylation of pro-mitotic substrates (e.g., CDC25C). We used CDC25C phosphorylation (Wang et al., 2007) as a marker for MAPK activity in the progression to mitosis. We observed that CDC25C modification increases in HRG-treated cells (see Figures 4A and S6C for MCF-7 and RPE-1 cells, respectively), even in the presence of DNA damage, while no phosphorylation is observed after DNA damage alone. Moreover, CDC25C phosphorylation is decreased by U0126 treatment, confirming its dependence on MAPK (Figure S5H). Furthermore, consistent with our hypothesis, HRG increases the expression of the pro-mitotic factors PLK1 and CYCLIN B1, even in the presence of DNA damage in both asynchronous and G2-synchronized MCF-7 cells (Figures 4B–4F), and a similar increase in PLK1 levels is observed in synchronized RPE-1 cells (Figures S6A and S6B), whereas no increase is seen in the presence of EGF (Figure S5G). Together with the observed decrease in p53 levels induced by HRG, this confirms that sustained MAPK activation weakens the G2 checkpoint, thereby permitting cells to progress into mitosis with unresolved DNA damage, as reported previously (Liang et al., 2014) and confirmed in Figure 5A by γH2AX staining. Thus, our findings define an unnoticed mechanism wherein dynamic changes in MAPK activity maintain an optimal balance between pro-survival and pro-arrest signals during the DNA damage response. Sustained MAPK activity imbalances this mechanism, enfeebling damage surveillance via the G2 checkpoint.

## Discussion

Cell fate choices at cell-cycle checkpoints are critical for the maintenance of tissue homeostasis and the genomic integrity of the cell. However, it is now apparent that in mammalian cells, DNA damage checkpoints in the G1 and G2 phases of the cell cycle are not infallible (Deckbar et al., 2011, Löbrich and Jeggo, 2007), but often permit cell-cycle progression in the presence of DNA damage. It is conceivable that the fidelity of DNA damage checkpoints has evolved to balance the requirements that minimize the probability of the emergence of genomic instability, and to maximize at the same time the homeostatic requirements for tissue maintenance.

In this work, we demonstrate that MAPK signaling is activated by the acute exposure of cells to DNA damage, triggering previously unnoticed pulses of ERK activity that are reminiscent of the pulsatile behavior of p53. We find that the transient nature of MAPK activation and p53 expression cooperate to control specific transcriptional programs, moderating the p53 response and permitting the continued accumulation of pro-mitotic factors such as PLK1 and CYCLIN-B1, even during checkpoint enforcement. These data exemplify that the dynamic interaction between MAPK and p53 signaling is indispensable to poise cells in an ambiguous state (Lahav, 2004); both MAPK and p53 are conducive to DNA damage repair, cell death, and senescence and to cell-cycle progression (Figure 5B). The abrogation of MAPK signaling enhances checkpoint stringency, whereas its sustained stimulation with HRG or light-inducible RAF increases checkpoint negligence. Mechanistically, sustained MAPK signaling during checkpoint activation results in the MAPK/ERK-mediated phosphorylation of CDC25C, leading to the accumulation of pro-mitotic factors such as CYCLIN B1 and PLK1. Notably, we did not detect significant changes in the length of G2 in NCS- and HRG- treated cells, indicating that a sustained mitogenic signal does not shorten G2, but instead increases the probability that cells progress to mitosis rather than undergo prolonged cell-cycle arrest and/or cell death. Together with previous reports that not only demonstrate that signaling dynamics mediate cell fate determination (Batchelor et al., 2011, Purvis et al., 2012, Aoki et al., 2013, Santos et al., 2007) but also their relevance as actionable drug targets (Chen and Lahav, 2016, Santos et al., 2007), our findings highlight the importance of investigating cellular decisions together with temporal changes in the underlying biochemical networks.

We find that both MCF-7 and RPE1 cells exhibit similar overall dynamics for p53 expression and ERK activation after DNA damage. We detect some differences that can plausibly be attributed to well-known variations in p53 dynamics after different genotoxic challenges (Geva-Zatorsky et al., 2006, Wu et al., 2017). Thus, our results are consistent with the notion that the concurrent activation of apparently opposing signals is an important emerging feature characterizing cell fate choices at cell-cycle checkpoints. Our results also suggest that the strength of these opposing signals and their dynamics may vary between cell types and type of damage to fine-tune the stringency of checkpoint enforcement in a context-dependent manner.

Our proposal that a competing-networks model dictates cell fate choices following damage-induced G2 checkpoint activation attracts interesting parallels (Figure 5C). For instance, such a competing-networks model has been invoked to explain the balance between exit from mitosis and death in mitosis (Topham et al., 2015), wherein the rate of CYCLIN B1 degradation appears to compete against activation of the pro-apoptotic machinery, deciding cell fate at the spindle assembly checkpoint. Similarly, cell fate at the G2 checkpoint depends on background PLK1 activation (Liang et al., 2014, Jaiswal et al., 2017), and the opposing activities of p53 and inhibitor-of-apoptosis proteins (IAPs) (Paek et al., 2016) to set a balance between cell death and survival.

Notably, our findings demonstrate that the MAPK-dependent mechanism activated by DNA damage remains sensitive to extracellular cues such as growth factors, enabling tissue homeostasis to balance optimally with the tumor-suppressive function of the G2 checkpoint. The importance of these observations is highlighted by the possibility that this mechanism could be hijacked by oncogenic activation of RAS or other mutations within the RAS-dependent signal transduction networks (e.g., in EGF receptor [EGFR] or RAF). By weakening the G2 checkpoint via sustained MAPK activation, oncogene-driven proliferation may increase genome instability to promote cellular transformation.

## STAR★Methods

### Key Resources Table

**Table:** 

REAGENT or RESOURCE	SOURCE	IDENTIFIER
**Antibodies**		

p53 (DO-1)	Santa Cruz	sc-126; RRID:AB_628082
phospho-p44/42 MAPK (E10)	Cell Signaling	#9106; RRID:AB_331768
p44/42 MAPK	Cell Signaling	# 9102S; RRID:AB_330744
Beta-actin	Sigma-Aldrich	A2228; RRID:AB_476697
ATM	Sigma-Aldrich	A1106; RRID:AB_796190
phospho-ATM (Ser1981) [EP1890Y]	AbCam	ab81292; RRID:AB_1640207
phospho-c-Fos (Ser374)	Calbiochem	NA
c-Fos	Santa Cruz	sc-166940; RRID:AB_10609634
phospho-cdc25C (Thr48)	Cell Signaling	#12028; RRID:AB_2797802
Cdc25c (C20)	Santa Cruz	sc-327; RRID:AB_2075277
EGFP (JL-8)	Clontech	632380; RRID:AB_10013427
c-Raf	Cell Signaling	#9422; RRID:AB_390808
Plk1	Invitrogen	#33-1700; RRID:AB_2533104
Cyclin B1	Santa Cruz	sc-245; RRID:AB_627338
phospho-MAPK substrates motif PXpTP	Cell Signaling	#14378; RRID:AB_2798468
IR800 CW	Li-Cor	926-32210; RRID:AB_621842
IR 680 RD	Li-Cor	926-68073; RRID:AB_10954442
gH2AX antibody	Calbiochem	05-636; RRID:AB_309864

**Bacterial and Virus Strains**		

One Shot TOP10 Chemically Competent *E. coli*	Thermo Fisher Scientific (Life Technologies)	C404010

**Chemicals, Peptides, and Recombinant Proteins**		

InSolution-U0126	Calbiochem	662009
recombinant HRG rhNRG1Beta/HRG1-Beta 1	R&D systems	396-HB
EGF	Sigma-Aldrich	E9644
Ku-55933	Selleck Chemicals	S1092
Doxorubicin	Sigma-Aldrich	D1515
Neocarzinostatin	Sigma-Aldrich	N9162-100UG
Lipofectamine 2000 Transfection Reagent	ThermoFisher	Cat# 11668019

**Critical Commercial Assays**		

First Strand cDNA Synthesis Kit for RT-PCR (AMV)	Roche	04896866001
LightCycler 480 SYBR Green Master Mix	Roche	04707516001
RNeasy mini kit	QIAGEN	74104
Senescence β-Galactosidase Staining Kit	Cell Signaling	#9860

**Experimental Models: Cell Lines**		

MCF-7	Cancer Research UK Cell Services	NA
hTERT-RPE 1	ATCC	CRL-4000

**Oligonucleotides**		

RT-PCR primer: EGR1 FP: CTT CAA CCC TCA GGC GGA CA RP: GAA AAG CGG CCA GTA TAG GT	Sigma-Aldrich	NA
RT-PCR primer:JUN B FP: CTG GTG GCC TCT CTC TAC ACG RP: CCC GCG GGG GTA AAA GTA CTG	Sigma-Aldrich	NA
RT-PCR primer:FOS FP:AGG AGG GAG CTG ACT GAT ACA CTP:TTT CCT TCT CCT TCA GCA GGT T	Sigma-Aldrich	NA
RT-PCR primer:DUSP1 FP:TGT GGA GGA CAA CCA CAA GG RP: AAA CTC AAA GGC CTC GTC CA	Sigma-Aldrich	NA
RT-PCR primer: CTGF FP:AGC CGC CTG TGC ATG GT RP: GGG AGT ACG GAT GCA CTT TTT G	Sigma-Aldrich	NA
RT-PCR primer: p21 FP:TGA GCC GCG ACT GTG ATGRP:GTC TCG GTG ACA AAG TCG AAG TT	Sigma-Aldrich	NA
RT-PCR primer:GADD45a FP:CGT TTT GCT GCG AGA ACG AC RP:GAA CCC ATT GAT CCA TGT AG	Sigma-Aldrich	NA
RT-PCR primer:XPC FP:TGA GAC CAT ACC AGA GCC CA RP:GGG CAT ACA GAG GGT GGT TC	Sigma-Aldrich	NA
RT-PCR primer:GAPDH FP:TGA GCT TGA CAA AGT GGT CG RP:GTC AGT GGT GGA CCT GAC CT	Sigma-Aldrich	NA

**Recombinant DNA**		

pEKAREV (3560NES)	Gift from Prof. Michiyuki Matsuda and Dr. Aoki Kazuhiro	Komatsu et al., 2011
pCX4puro-CRY2-cRaf	Gift from Prof. Michiyuki Matsuda and Dr. Aoki Kazuhiro	Aoki et al., 2013
pCX4neo-CIBN-EGFP-KRasCT	Gift from Prof. Michiyuki Matsuda and Dr. Aoki Kazuhiro	Aoki et al., 2013
pcDNA3.1/nV5-DEST-mCherry-H2B	Gift from Dr. Rob Mahen	MRC-CU

**Software and Algorithms**		

GraphPad Prism 5	GraphPad	https://www.graphpad.com/scientific-software/prism/
Image Studio	LI-COR	https://www.licor.com/bio/image-studio/
MATLAB	Mathworks	https://www.mathworks.com

### Lead Contact and Materials Availability

Further information and requests for resources and reagents should be directed to and will be fulfilled by the Lead Contact, Alessandro Esposito (ae275@mrc-cu.cam.ac.uk). All unique/stable reagents generated in this study are available from the Lead Contact without restriction.

### Experimental Model and Subject Details

#### Human Cell lines

MCF-7 (Cancer Research UK Cell Services) and hTERT-RPE (ATCC, CRL-4000) cell lines were grown in standard DMEM and DMEM/F12, respectively, supplemented with 10% FCS. MCF-7 cell line stably expressing p53 fused with the Venus fluorescent protein (Purvis et al., 2012) was a gift from Prof. Galit Lahav and were used for the measurement of single cell G2 and M cell cycle traces. MCF-7 cells stably expressing the EKAREV sensor (MCF7-EKAREV) were derived from parental MCF-7 cells transfected with pEKAREV, then sorted into single cell (by single cell sorting) clones utilizing equal fluorescence emission of the donor and acceptor fluorophores, YPet and ECFP, to check for expression of a putatively intact sensor. MCF7-EKAREV transfected cells were validated by western blotting and imaging in the presence of EGF and HRG. MCF7-EKAREV cells were further modified to stably express mCherry-H2B as nuclear marker, wherein cells with the integrated plasmid were selected utilizing single cell sorting to select the final clone that exhibited medium intensities for all the three fluorophores. The light-inducible CRY2-cRaf MCF-7 stable cell line (MCF7-OptoRaf) was derived from the MCF-7 parental line by retrovirally-mediated transduction of pCX4puro-CRY2-cRaf and pCX4neo-CIBN-EGFP-KRasCT followed by selection of a single clone using Puromycin and Neomycin selection. All cell lines were maintained at 37°C and 5% CO_2_.

The identity of cell lines was confirmed by STR profiling utilizing the CRUK Cambridge Institute facilities. For cell cycle synchronization, cells were cultured in DMEM supplemented with 2mM thymidine for 16 hours. Cells were then washed 3 times with PBS and released in DMEM for 8 hours. A second identical thymidine block and release was subsequently applied typically obtaining > 50% MCF-7 in the S-phase once cells were released from the block and allowed to progress into the subsequent cell cycle phases. In average cells were in G2 8 hr post-release; experiments were carried out at the time points indicated in the main text and figures. Mechanical stimulation or changes in the concentration of growth factors, such as the addition of fresh media, can activate MAPK signaling. Therefore, to avoid artifacts caused by the addition of drugs during the experiments, cells were grown for at least 12 hours in multiwell or single plates (depending on the assay) to allow accomplishment of steady state activity of ERK signaling. Subsequently, small volumes of drugs or appropriate vehicle controls (0.8 μl into 2 ml) were added to the media that was already present in the wells (Figure S1B). The plates, which included also the controls, were then gently swirled by hand. This procedure provided us well-controlled and drug-specific responses in MAPK activity.

### Method Details

#### Plasmids

The plasmids encoding the FRET-based biosensor reporting for ERK activity (Komatsu et al., 2011) pEKAREV (3560NES) and the light-inducible RAF (Aoki et al., 2013) (pCX4puro-CRY2-cRaf and pCX4neo-CIBN-EGFP-KRasCT) were kindly provided by Prof. Michiyuki Matsuda and Dr. Aoki Kazuhiro. The pCX4 plasmids used to develop the optogenetic tools were provided by Dr. Jun-ichi Miyazaki (Niwa et al., 1991). The plasmid encoding for mCherry fused to histone H2B (pcDNA3.1/nV5-DEST-mCherry-H2B) was a gift by Dr. Rob Mahen.

#### Western blotting

Cells were lysed on the plate in RIPA buffer in the presence of protease and phophatase Inhibitors; protein concentrations were estimated by BCA assay. The lysates were separated using 4%–12% NuPAGE Bis Tris SDS-PAGE gels (Invitrogen) and transferred to Amersham Protran nitrocellulose membrane (Catalogue number 10600008), followed by blocking with appropriate blocking buffer (5% milk or 5% BSA in Tris Buffered Saline-Tween). The membranes were incubated with the following primary antibodies in 5% milk blocking solution, unless otherwise indicated: mouse p53 (DO-1, Santa Cruz, 1:500), mouse phospho-p44/42 MAPK (Erk1/2, Thr202/Tyr204) (E10, Cell Signaling, 1:1000), rabbit p44/42 MAPK (Cell Signaling, 1:1000), mouse beta-actin (Sigma,1:10,000), mouse ATM (Monoclonal, Sigma, 1:500), rabbit phospho-ATM (Ser1981) (AbCam,1:1000, Rabbit), mouse phospho-c-Fos (Ser374) (Calbiochem, 1:500 in 5% BSA), mouse c-Fos (Santa Cruz, 1:500 in 5% BSA), rabbit phospho-cdc25C (Thr48) (Cell Signaling, 1:500 in 5% BSA), rabbit Cdc25c (C20, Santa Cruz, 1:500), mouse EGFP (JL-8, Clontech,1:1000), mouse c-Raf (Cell Signaling, 1:1000), mouse Plk1 (Invitrogen, 1:1000), mouse Cyclin B1 (Santa Cruz, 1:1000), rabbit monoclonal phospho-MAPK substrates motif PXpTP (Cell Signaling 1:1000 in 5% BSA). Mouse and rabbit antibodies were detected with anti-mouse IR800 CW or anti-rabbit IR 680 RD (LI-COR antibody, 1:5000), respectively. Quantitative analysis of the western blots was achieved by imaging the membranes with the Odyssey scanner (LI-COR). All band intensities were analyzed using LI-COR Image Studio.

#### RT-PCR analysis

Cells were lysed and homogenized using the Qiashredder kit (QIAGEN, 79654), and RNA was isolated using the RNeasy mini kit (QIAGEN, 74104). 1 μg of RNA per sample was used to synthesize cDNA using the First Strand cDNA Synthesis Kit for RT-PCR (AMV) (Roche, 11483188001). The resulting 20μl cDNA samples were diluted to 50μl in RNase-free water. qRT-PCR was carried out in triplicates with LightCycler 480 SYBR Green Master Mix (Roche, 4707516001) using a LightCycler 480 instrument (Roche). Relative abundances were calculated as the ratio of 2^-ΔΔCT^ normalized to GAPDH. The following (forward and reverse)primer pairs were used for EGR1 (CTT CAA CCC TCA GGC GGA CA and GAA AAG CGG CCA GTA TAG GT), JUN B- (CTG GTG GCC TCT CTC TAC ACG and CCC GCG GGG GTA AAA GTA CTG), FOS (AGG AGG GAG CTG ACT GAT ACA CT and TTT CCT TCT CCT TCA GCA GGT T), DUSP1(TGT GGA GGA CAA CCA CAA GG and AAA CTC AAA GGC CTC GTC CA), CTGF (AGC CGC CTG TGC ATG GT and GGG AGT ACG GAT GCA CTT TTT G), p21 (TGA GCC GCG ACT GTG ATG and GTC TCG GTG ACA AAG TCG AAG TT), GADD45a (CGT TTT GCT GCG AGA ACG AC and GAA CCC ATT GAT CCA TGT AG), XPC (TGA GAC CAT ACC AGA GCC CA and GGG CAT ACA GAG GGT GGT TC), and GAPDH (TGA GCT TGA CAA AGT GGT CG and GTC AGT GGT GGA CCT GAC CT).

#### Immunofluorescence assays

Cells were plated on coverslips in 6 well plates, subsequently synchronized by double-thymidine block, and released into fresh media. After 6 hours, cells were treated with NCS, U0126, EGF or HRG. At the same time, nocadazole (100 ng/ml) was added to prevent cells from completing mitosis. After 4 hours, cells were fixed with ice cold methanol for 15 minutes. DNA damage was then probed with a γH2AX antibody (mouse,1:1000, Calbiochem) and Goat ant-mouse Alexa Fluor 488 secondary antibody (1:500, Invitrogen). Images were acquired with a Leica SP5 confocal microscope, utilizing a HCXPLAPOCS 40x oil-immersion objective and analyzed with the Cellomics software.

#### Live cell imaging and image analysis

Cells were plated on 2- or 4- wells Lab-Tek chambers (Thermo Fisher Scientific, Cat. No. 155380 2-wells and 155383 4-wells) washed and kept in DMEM without phenol red (Invitrogen, Cat. No. 31053028) supplemented with 10% FCS. Images were acquired with a motorized Leica DMI6000B microscope, using a HCX PL APO 40X/0.85 dry objective equipped with a fast switching filter-wheel dedicated to sensitized FRET imaging and a Photometrics Evolve 512 EMCCD camera. The same system was used to measure brightfield images. Each image was captured after every 3 minutes, with a minimum of 7 fields of view for each condition. Treatments were performed on stage after the setup of the imaging session, just prior to the start of imaging. For assays with synchronized cells, imaging started 6 hours after release from thymidine block with treatments as indicated in the main text and figures. According to flow cytometry (Figure S1A), this timing ensured that cells were tracked with live cell imaging starting from late S-phase or early G2-phase. Only cells that entered the first mitosis post-treatment were considered for analysis to avoid including cells that might have passed through multiple stages of the cell cycle. Moreover, only cells that entered mitosis within a maximum of 24 or 12 hr were considered for analysis to avoid spurious results from those cells that might be released late from G1. 24 hr was used for experiments with NCS and U0126, shortened to 12 hr for HRG and OptoRAF to account for possible agonists of MAPK signaling on G1/S transition.

The EKAREV FRET sensor was excited with a mercury short arc lamp using a 463 ± 20 nm band-pass filter. Fluorescence was separated with a dichroic mirror (455 nm cut-off wavelength) and fluorescence emitted from the ECFP and YPet were collected sequentially using the fast switching filter-wheel equipped with 480 ± 40 nm and 535 ± 30 nm band-pass filters, respectively. The ratio of fluorescence intensities emitted from YPet and ECFP (YPet/ECFP) was used as a surrogate measure of ERK activity (Komatsu et al., 2011). mCherry-H2B fluorescence was acquired with the standard N3/red filter from Leica. Typical acquisition times were around 120ms, 120ms and 300ms for YPET, ECFP and mCherry, respectively. Data analysis was performed with an in-house developed MATLAB toolbox (Mathworks) similar to what we have previously described (Liang et al., 2014). Briefly, data was imported in MATLAB with the Bio-Formats toolbox developed by the Open Microscopy Environment network and a gentle median filtering with a 2x2 kernel was applied to denoise images. The nuclear marker (mCherry-H2B) was used for segmentation and tracking; unsupervised segmentation was performed by simple intensity thresholding followed by watershedding; tracking was performed by inheriting cell identifiers from one frame (*t*) to the following (*t+1*), identifying cells at *t+1* with maximum area overlap with cells at time *t*. Very small objects (< 100 pixels in area, i.e., ∼30 μm^2^) were discarded to remove segmented cellular debris. The results of this fast unsupervised step were manually curated with a graphic user interface that allowed a user to reassign wrongly identified cells or delete cells which traces were unreliable (e.g., cells migrating outside the boundaries of a field of view and reclassified ambiguously with an adjacent cell). Only the remaining, accurately segmented and tracked non-mitotic cells, were carried over to the final analysis. The YPet and ECFP ratio was determined as the ratio between the mean intensities of rings 1 pixel away from the segmented nuclei and 5 pixel thick, only on pixels belonging to the watershedded region of the analyzed cell. All traces that were too short (< 10hrs), that exhibited contiguous gaps longer than three frames or total gaps > 5% of the traces were pruned automatically. Where present, short gaps in traces where healed applying a local median filter with a kernel of 6 time points. FRET traces for treated and untreated cells were first normalized to their time zero values. In order to compute confidence intervals, we then normalized all traces to the average trace of the untreated cells. The bootstrapped average and 95% confidence intervals of both standardized groups were then calculated resampling the distributions 10,000 times. This procedure allowed us to compute a standardized average response of ERK activities after DNA damage providing meaningful errors for both the untreated (normalized to one at each time point) and treated samples (normalized to untreated). The data and the MATLAB code we have used to generate the representations shown are provided as supporting materials.

#### Chemicals

The MEK inhibitor InSolution-U0126 (662009, Calbiochem), recombinant HRG rhNRG1Beta/HRG1-Beta 1 (396-HB, R&D systems), EGF (Sigma), the ATM inhibitor Ku-55933 (S1092, Selleck Chemicals), Doxorubicin (Sigma, D1515) and the radiomimetic drug Neocarzinostatin (N9162-100UG, Sigma) were added to full media at the concentration described in the main text.

#### siRNA-mediated protein knock-downs

Cells were plated in 6 well multi-plates in standard DMEM supplemented with 10% FCS; the following day, cells were transfected with either 20nM of pooled siRNA targeting p53 (ON-TARGET plus Human TP53 (7157) siRNA, Thermo Scientific) or a control siRNA against Luciferase (MWG) using Lipofectamine 2000, following manufacturer’s protocol. 48 hours post-transfection, cells were treated at the desired time points with NCS as indicated in the main text, and then harvested for subsequent western blot analysis.

#### Beta gal Assay

After double-thymidine block, cells were plated in 6 multi-well plates in DMEM supplemented with 10% FCS. G2-synchronized cells were then treated with NCS, U0126, EGF and HRG: EGF single pulse at 1 hr, multiple EGF pulses at 1 and 3 hours, U0126 pre-treatment for 30 minutes before NCS addition and HRG at the time of NCS addition. Cells were then washed with fresh media after 12 hours. Cells were fixed and stained after four days as per manufacturer protocol (Senescence β-Galactosidase Staining Kit #9860, Cell Signaling), and scored manually on a standard wide field microscope.

#### Cell-Titer Blue Assay

RPE-1 cells were plated in 96-well plate at a density of 2000cells/well and synchronized with a single-thymidine block. 6 hours after release in fresh media, cells were treated with or without DNA damage, and HRG, EGF and U0126, in triplicates. After 3 days, cells were incubated with 20 μL of Cell-Titer Blue reagent (Promega, catalog number G8080) for 4 hours and the fluorescent values were measured at 560/590nm using Tecan Infinite m200 plate reader.

### Quantification and Statistical Analysis

#### Statistical analyses

All the reported multiple comparisons were performed as one-way ANOVA with Newman-Keuls multiple comparison test. Symbols to indicate statistical significance within figures related to p values thresholds of 0.05 (^∗^), 0.01 (^∗∗^) and 0.001 (^∗∗∗^) as evaluated by GraphPad for post-test pairwise comparisons. For ANOVA tests, the exact p values on testing if all the means come from the same distribution are shown in Table S1. Here, also the p value for a two-tail-test used in Figure 4B is shown.

#### Western Blotting

Quantitative analysis of the western blots was achieved by imaging the membranes with the Odyssey scanner (LI-COR). All band intensities were analyzed using LI-COR Image Studio.

### Data and Code Availability

The published article includes all datasets analyzed during this study. The code used to generate Figure 2C is provided in Data S1.

## Supplemental Information

Document S1. Figures S1–S6 and Table S1

Data S1. Data and MATLAB Code Used to Generate Figure 2C

Document S2. Article plus Supplemental Information

## References

[bib1] Aoki K., Kumagai Y., Sakurai A., Komatsu N., Fujita Y., Shionyu C., Matsuda M. (2013). Stochastic ERK activation induced by noise and cell-to-cell propagation regulates cell density-dependent proliferation. Mol. Cell.

[bib2] Batchelor E., Mock C.S., Bhan I., Loewer A., Lahav G. (2008). Recurrent initiation: a mechanism for triggering p53 pulses in response to DNA damage. Mol. Cell.

[bib3] Batchelor E., Loewer A., Mock C., Lahav G. (2011). Stimulus-dependent dynamics of p53 in single cells. Mol. Syst. Biol..

[bib4] Behrens A., van Deursen J.M., Rudolph K.L., Schumacher B. (2014). Impact of genomic damage and ageing on stem cell function. Nat. Cell Biol..

[bib5] Chen S.H., Lahav G. (2016). Two is better than one; toward a rational design of combinatorial therapy. Curr. Opin. Struct. Biol..

[bib6] Cohen-Saidon C., Cohen A.A., Sigal A., Liron Y., Alon U. (2009). Dynamics and variability of ERK2 response to EGF in individual living cells. Mol. Cell.

[bib7] Davies H., Bignell G.R., Cox C., Stephens P., Edkins S., Clegg S., Teague J., Woffendin H., Garnett M.J., Bottomley W. (2002). Mutations of the BRAF gene in human cancer. Nature.

[bib8] Deckbar D., Jeggo P.A., Löbrich M. (2011). Understanding the limitations of radiation-induced cell cycle checkpoints. Crit. Rev. Biochem. Mol. Biol..

[bib9] Ermolaeva M.A., Dakhovnik A., Schumacher B. (2015). Quality control mechanisms in cellular and systemic DNA damage responses. Ageing Res. Rev..

[bib10] Favata M.F., Horiuchi K.Y., Manos E.J., Daulerio A.J., Stradley D.A., Feeser W.S., Van Dyk D.E., Pitts W.J., Earl R.A., Hobbs F. (1998). Identification of a novel inhibitor of mitogen-activated protein kinase kinase. J. Biol. Chem..

[bib11] Gerarduzzi C., de Polo A., Liu X.S., El Kharbili M., Little J.B., Yuan Z.M. (2016). Human epidermal growth factor receptor 4 (Her4) Suppresses p53 Protein via Targeting the MDMX-MDM2 Protein Complex: IMPLICATION OF A NOVEL MDMX SER-314 PHOSPHOSITE. J. Biol. Chem..

[bib12] Geva-Zatorsky N., Rosenfeld N., Itzkovitz S., Milo R., Sigal A., Dekel E., Yarnitzky T., Liron Y., Polak P., Lahav G., Alon U. (2006). Oscillations and variability in the p53 system. Mol. Syst. Biol..

[bib13] Hickson I., Zhao Y., Richardson C.J., Green S.J., Martin N.M., Orr A.I., Reaper P.M., Jackson S.P., Curtin N.J., Smith G.C. (2004). Identification and characterization of a novel and specific inhibitor of the ataxia-telangiectasia mutated kinase ATM. Cancer Res..

[bib14] Jaiswal H., Benada J., Müllers E., Akopyan K., Burdova K., Koolmeister T., Helleday T., Medema R.H., Macurek L., Lindqvist A. (2017). ATM/Wip1 activities at chromatin control Plk1 re-activation to determine G2 checkpoint duration. EMBO J..

[bib15] Komatsu N., Aoki K., Yamada M., Yukinaga H., Fujita Y., Kamioka Y., Matsuda M. (2011). Development of an optimized backbone of FRET biosensors for kinases and GTPases. Mol. Biol. Cell.

[bib16] Krenning L., Feringa F.M., Shaltiel I.A., van den Berg J., Medema R.H. (2014). Transient activation of p53 in G2 phase is sufficient to induce senescence. Mol. Cell.

[bib17] Lahav G. (2004). The strength of indecisiveness: oscillatory behavior for better cell fate determination. Sci. STKE.

[bib18] Lahav G. (2008). Oscillations by the p53-Mdm2 feedback loop. Adv. Exp. Med. Biol..

[bib19] Lahav G., Rosenfeld N., Sigal A., Geva-Zatorsky N., Levine A.J., Elowitz M.B., Alon U. (2004). Dynamics of the p53-Mdm2 feedback loop in individual cells. Nat. Genet..

[bib20] Lau A., Swinbank K.M., Ahmed P.S., Taylor D.L., Jackson S.P., Smith G.C., O’Connor M.J. (2005). Suppression of HIV-1 infection by a small molecule inhibitor of the ATM kinase. Nat. Cell Biol..

[bib21] Liang H., Esposito A., De S., Ber S., Collin P., Surana U., Venkitaraman A.R. (2014). Homeostatic control of polo-like kinase-1 engenders non-genetic heterogeneity in G2 checkpoint fidelity and timing. Nat. Commun..

[bib22] Löbrich M., Jeggo P.A. (2007). The impact of a negligent G2/M checkpoint on genomic instability and cancer induction. Nat. Rev. Cancer.

[bib23] Loewer A., Batchelor E., Gaglia G., Lahav G. (2010). Basal dynamics of p53 reveal transcriptionally attenuated pulses in cycling cells. Cell.

[bib24] Mandriota S.J., Buser R., Lesne L., Stouder C., Favaudon V., Maechler P., Béna F., Clément V., Rüegg C., Montesano R., Sappino A.P. (2010). Ataxia telangiectasia mutated (ATM) inhibition transforms human mammary gland epithelial cells. J. Biol. Chem..

[bib25] Murphy L.O., Smith S., Chen R.H., Fingar D.C., Blenis J. (2002). Molecular interpretation of ERK signal duration by immediate early gene products. Nat. Cell Biol..

[bib26] Nakakuki T., Birtwistle M.R., Saeki Y., Yumoto N., Ide K., Nagashima T., Brusch L., Ogunnaike B.A., Okada-Hatakeyama M., Kholodenko B.N. (2010). Ligand-specific c-Fos expression emerges from the spatiotemporal control of ErbB network dynamics. Cell.

[bib27] Niwa H., Yamamura K., Miyazaki J. (1991). Efficient selection for high-expression transfectants with a novel eukaryotic vector. Gene.

[bib28] Paek A.L., Liu J.C., Loewer A., Forrester W.C., Lahav G. (2016). Cell-to-Cell Variation in p53 Dynamics Leads to Fractional Killing. Cell.

[bib29] Porter J.R., Fisher B.E., Batchelor E. (2016). p53 Pulses Diversify Target Gene Expression Dynamics in an mRNA Half-Life-Dependent Manner and Delineate Co-regulated Target Gene Subnetworks. Cell Syst..

[bib30] Povirk L.F. (1996). DNA damage and mutagenesis by radiomimetic DNA-cleaving agents: bleomycin, neocarzinostatin and other enediynes. Mutat. Res..

[bib31] Purvis J.E., Karhohs K.W., Mock C., Batchelor E., Loewer A., Lahav G. (2012). p53 dynamics control cell fate. Science.

[bib32] Santos S.D., Verveer P.J., Bastiaens P.I. (2007). Growth factor-induced MAPK network topology shapes Erk response determining PC-12 cell fate. Nat. Cell Biol..

[bib33] Shaltiel I.A., Krenning L., Bruinsma W., Medema R.H. (2015). The same, only different - DNA damage checkpoints and their reversal throughout the cell cycle. J. Cell Sci..

[bib34] Shankaran H., Ippolito D.L., Chrisler W.B., Resat H., Bollinger N., Opresko L.K., Wiley H.S. (2009). Rapid and sustained nuclear-cytoplasmic ERK oscillations induced by epidermal growth factor. Mol. Syst. Biol..

[bib35] Smith B.L., Bauer G.B., Povirk L.F. (1994). DNA damage induced by bleomycin, neocarzinostatin, and melphalan in a precisely positioned nucleosome. Asymmetry in protection at the periphery of nucleosome-bound DNA. J. Biol. Chem..

[bib36] Stagni V., Manni I., Oropallo V., Mottolese M., Di Benedetto A., Piaggio G., Falcioni R., Giaccari D., Di Carlo S., Sperati F. (2015). ATM kinase sustains HER2 tumorigenicity in breast cancer. Nat. Commun..

[bib37] Stewart-Ornstein J., Lahav G. (2017). p53 dynamics in response to DNA damage vary across cell lines and are shaped by efficiency of DNA repair and activity of the kinase ATM. Sci. Signal..

[bib38] Tentner A.R., Lee M.J., Ostheimer G.J., Samson L.D., Lauffenburger D.A., Yaffe M.B. (2012). Combined experimental and computational analysis of DNA damage signaling reveals context-dependent roles for Erk in apoptosis and G1/S arrest after genotoxic stress. Mol. Syst. Biol..

[bib39] Toettcher J.E., Loewer A., Ostheimer G.J., Yaffe M.B., Tidor B., Lahav G. (2009). Distinct mechanisms act in concert to mediate cell cycle arrest. Proc. Natl. Acad. Sci. USA.

[bib40] Topham C., Tighe A., Ly P., Bennett A., Sloss O., Nelson L., Ridgway R.A., Huels D., Littler S., Schandl C. (2015). MYC Is a Major Determinant of Mitotic Cell Fate. Cancer Cell.

[bib41] Wang R., He G., Nelman-Gonzalez M., Ashorn C.L., Gallick G.E., Stukenberg P.T., Kirschner M.W., Kuang J. (2007). Regulation of Cdc25C by ERK-MAP kinases during the G2/M transition. Cell.

[bib42] Wei F., Xie Y., Tao L., Tang D. (2010). Both ERK1 and ERK2 kinases promote G2/M arrest in etoposide-treated MCF7 cells by facilitating ATM activation. Cell. Signal..

[bib43] Wu M., Ye H., Tang Z., Shao C., Lu G., Chen B., Yang Y., Wang G., Hao H. (2017). p53 dynamics orchestrates with binding affinity to target genes for cell fate decision. Cell Death Dis..

[bib44] Yamamoto T., Ebisuya M., Ashida F., Okamoto K., Yonehara S., Nishida E. (2006). Continuous ERK activation downregulates antiproliferative genes throughout G1 phase to allow cell-cycle progression. Curr. Biol..

[bib45] Yan Y., Black C.P., Cowan K.H. (2007). Irradiation-induced G2/M checkpoint response requires ERK1/2 activation. Oncogene.

[bib46] Yang H.W., Chung M., Kudo T., Meyer T. (2017). Competing memories of mitogen and p53 signalling control cell-cycle entry. Nature.

[bib47] Zwang Y., Sas-Chen A., Drier Y., Shay T., Avraham R., Lauriola M., Shema E., Lidor-Nili E., Jacob-Hirsch J., Amariglio N. (2011). Two phases of mitogenic signaling unveil roles for p53 and EGR1 in elimination of inconsistent growth signals. Mol. Cell.

