# Supplementary material for: Pulsatile MAPK Signaling Modulates p53 Activity to Control Cell Fate Decisions at the G2 Checkpoint for DNA Damage
Source: Cell Rep. 2020 Feb 18;30(7):2083–2093.e5. doi: 10.1016/j.celrep.2020.01.074 (PMC7029415; doi:10.1016/j.celrep.2020.01.074)
Supplement: Document S1. Figures S1–S6 and Table S1 [file mmc1.pdf]

Cell Reports, Volume *30*

Supplemental Information

Pulsatile MAPK Signaling Modulates p53 Activity to Control Cell Fate Decisions at the G2 Checkpoint for DNA Damage

Siddharth De, Callum Campbell, Ashok R. Venkitaraman, and Alessandro Esposito

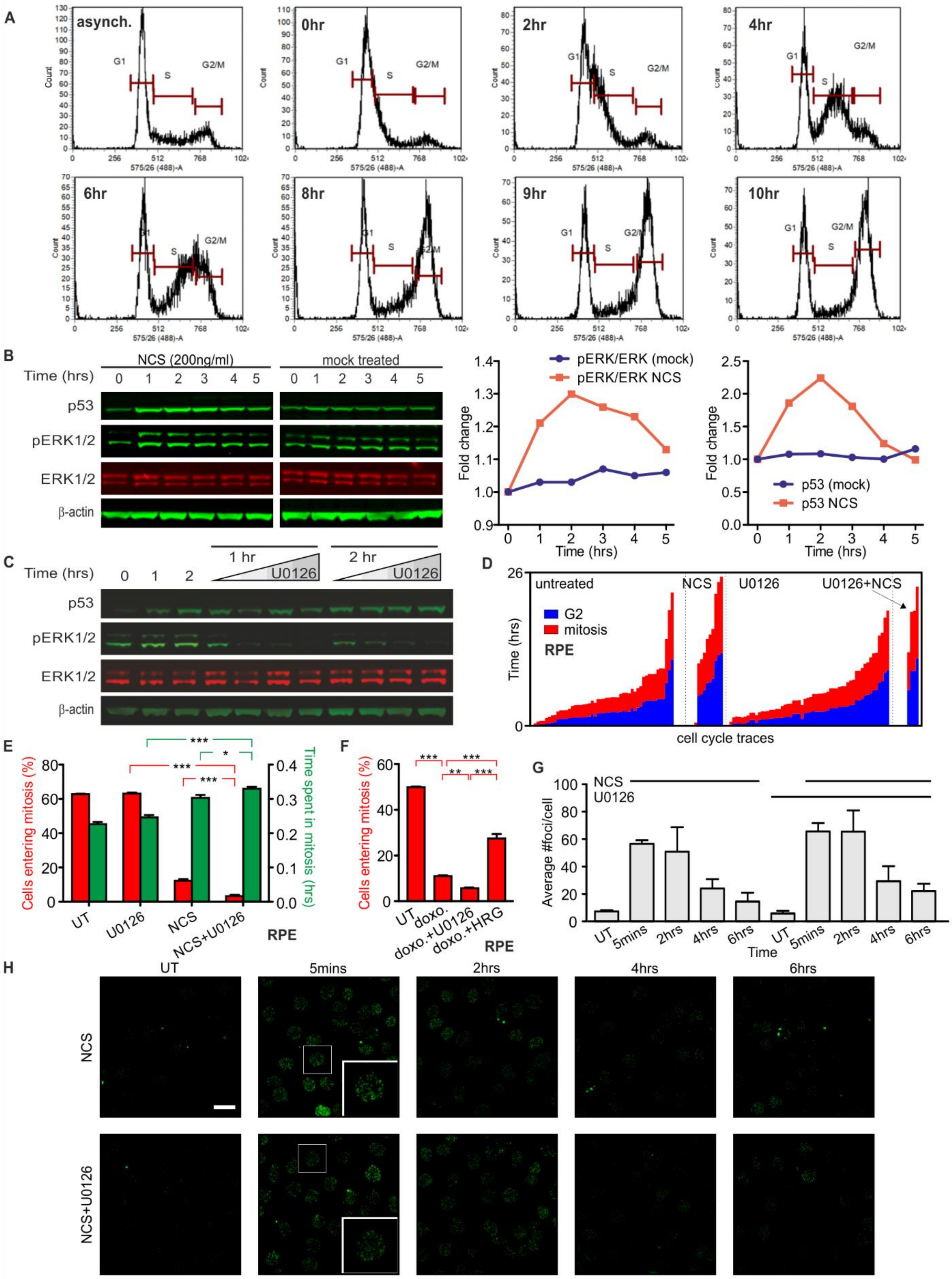

Figure S1. Validation of cell cycle synchronisation along with measurement of DNA damage in the absence or presence of U0126. DNA damage triggers p53 and MAPK oscillations that control checkpoint enforcement. Related to Figure 1.

(A) Cell-cycle analysis of MCF-7 cells done by flow cytometry, showing that at 8 hrs after the second release from Thymidine block, a large fraction of cells (41%) are in G2/M compared to asynchronous cells (15%), fraction further increasing to ~50% at subsequent timepoints.

(B) Representative Western Blot (n=2) of NCS treatment compared to a mock control (0.1% DMSO) showing that the pERK/ERK ratio and p53 expression do not change for unspecific chemical or mechanical stimuli that might occur during the addition of drugs to cell culture dishes. All the samples were run on the same gel, but the image was cropped to remove lysates from an unrelated experiment.

(C) Representative Western Blot (n=2) of MEK inhibitor U0126 titration (0.05 µM, 0.25 µM, 0.5 µM and 1 µM) in the presence of NCS.

(D) Cell fate traces of RPE cells either untreated or treated with 20 ng/ml (NCS), in the presence or absence of U0126 from one representative experiment of three repeats. In the presence of NCS, RPE cells exhibit a G2 arrest that is exarcebated by U0126, similarly to MCF-7 cells.

(E) Quantification of the single cell fate traces of RPE cells from (c) shows a significant decrease in cells entering mitosis when treated with NCS (20 ng) which gets further exacerbated when cells are treated with both NCS and U0126 (n=3).

(F) Quantification of number of synchronised RPE-1 cells entering mitosis when treated with Doxorubicin, along with U0126 and HRG. A significantly significant larger number of cells entered mitosis when cells were treated with HRG in the presence of Doxorubicin, while number of cells entering mitosis when treated with U0126 was significantly lower, indicating the enfeeblement of the G2 checkpoint (in the case of HRG) and higher stringency (in the case of U0126), upon the respective treatments (n=3).

(G) Quantification of DNA damage and repair, as measured by number of gammaH2AX staining, in MCF7 cells when treated with NCS and in the absence or presence of U0126. No significant difference in γH2AX staining is observed between cells treated with NCS alone or treated with both NCS and U0126 (n=3).

(H) Representative images from which the analysis shown in (c) was derived. Inset show a 2x magnification. Scale bar: 25µm

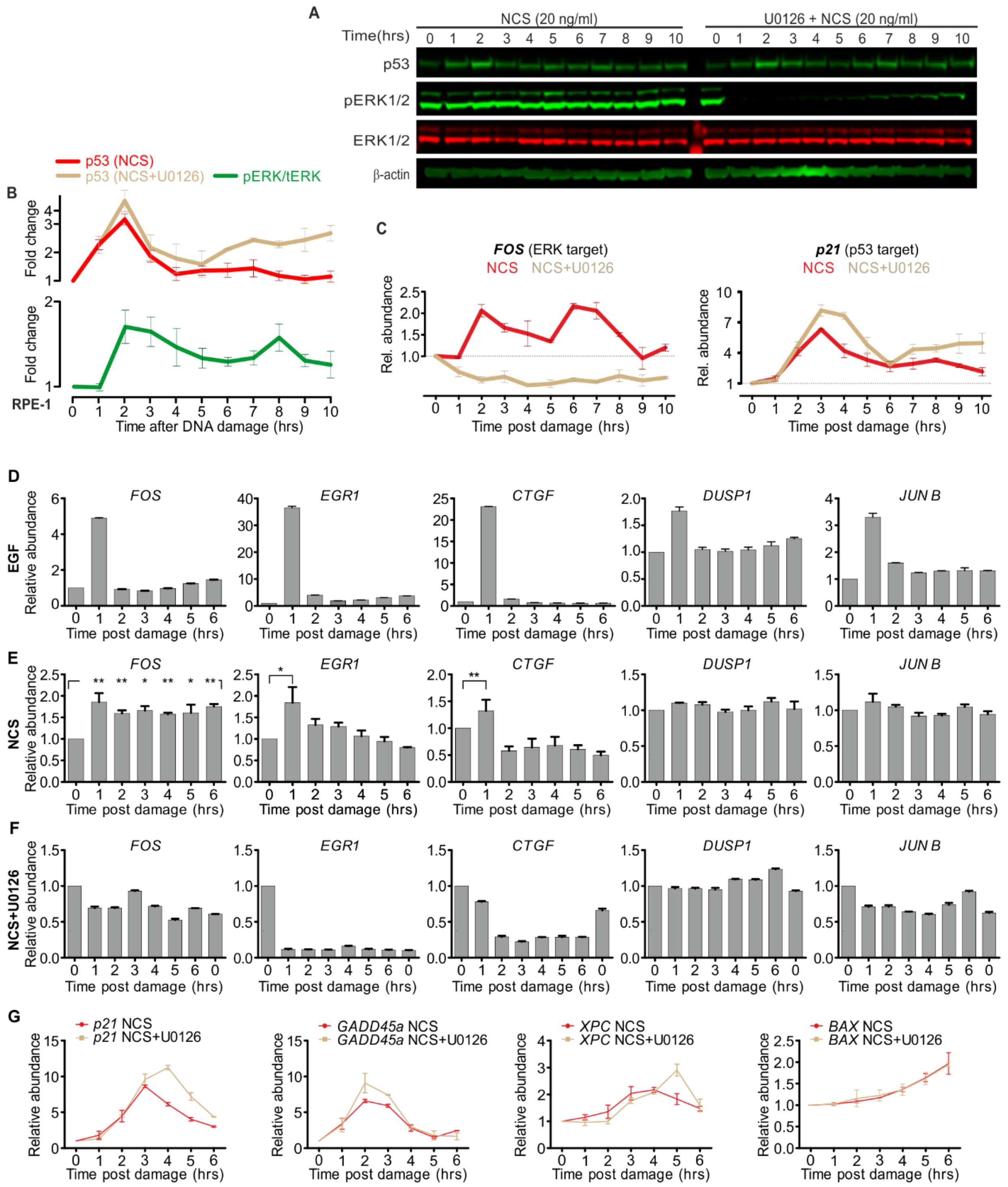

Figure S2. DNA-damage induced MAPK signalling reshapes cell transcriptional profiles. Related to Figure 1.

- **(A)** Representative Western Blot of three independent experiments for p53 and pERK dynamics in RPE cells treated with NCS (20 ng/ml).
- **(B)** Averages of p53 expression levels and pERK levels in RPE-1 cells post treatment with NCS. p53 expressions show a typical dynamic behaviour with two peaks with higher p53 expression levels when cells were treated with NCS and U0126. pERK/tERK levels also show a dynamic behaviour with peaks at approximately 2 hrs and 8 hrs, similar to those seen in MCF-7 cells (n=3)

(C) Expression profile of ERK target genes (c-FOS) and p53 target gene (p21) in RPE-1 cells when treated with NCS and with the concomitant inhibition of MAPK pathway inhibitor U0126 (n=3).

(D) Validation of ERK targets. qRT-PCR analysis (averages, n=3) of serum starved MCF7 cells treated with 100 ng/ml of EGF; this analysis confirms the transient response of ERK targets to EGF.

(E) Expression profile (mean± SEM) of ERK target genes (*FOS*, *EGR1*, *CTGF*, *DUSP1*, and *JUN B)* in the presence of DNA damage (n=3). Upon DNA damage, *FOS*, *EGR1* and *CTGF* transcripts are upreguated, at least transiently.

(F) With the concomitant inhibition of MAPK signaling using U0126, DNA-damage fails to trigger the response of these transcripts (n=3). Cells treated only with U0126 at 0 hrs were used to determine transcript levels after MAPK inhibition.

(G) Expression profile (n=3) of p53 target genes (*p21*, *GADD45a*, *XPC and BAX*) in the presence of DNA damage without (red curves) and with U0126 (yellow curves). Similarly to ERK, some p53 targets are insensitive to U0126 administration, but others such as p21, known to respond to pulsatile p53 responses, are significantly upregulated by addition of U0126 in the presence of DNA damage.

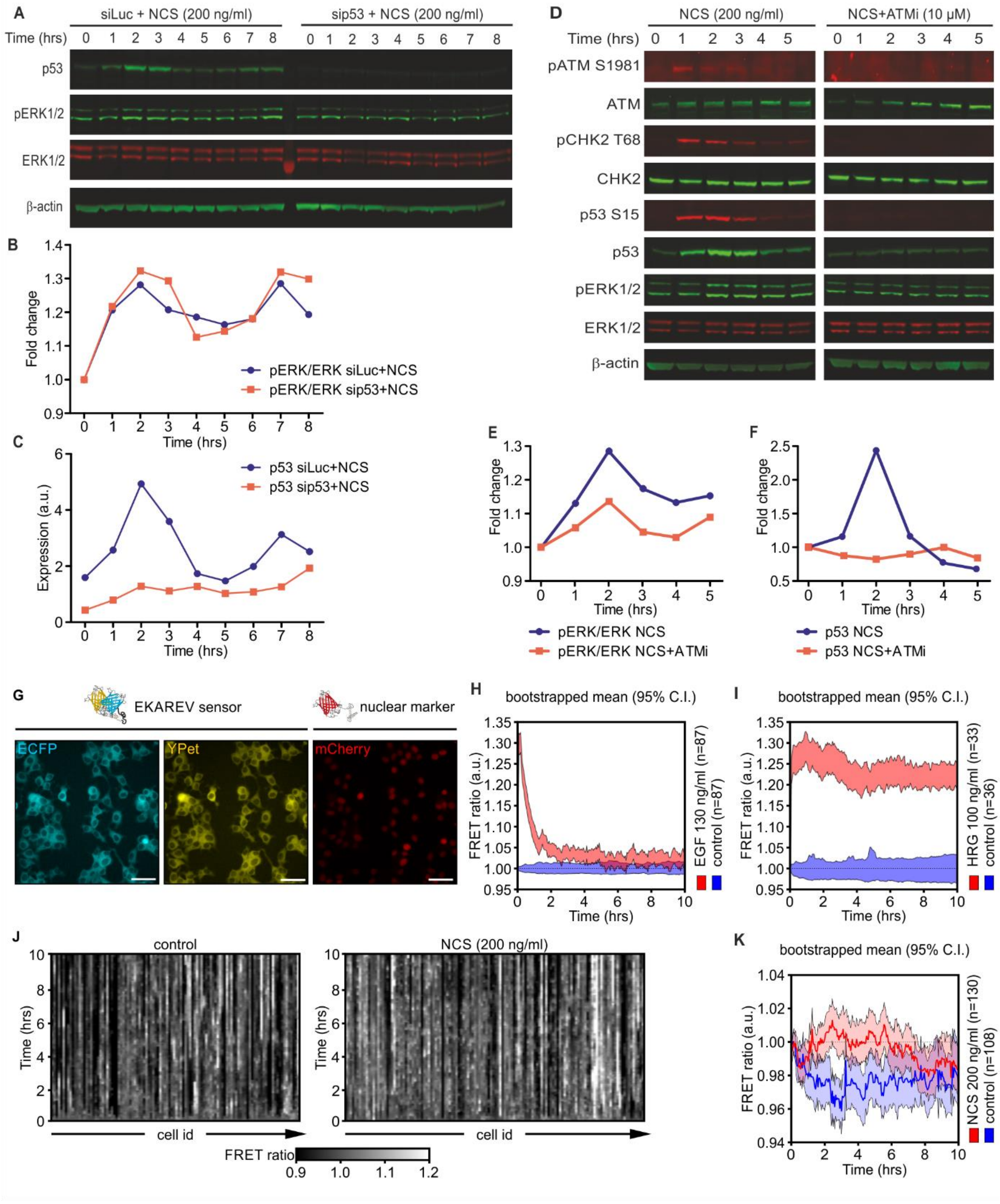

Figure S3. DNA damage triggers p53 and MAPK oscillations that control checkpoint enforcement. Related to Figures 2.

- **(A)** Representative Western Blot (n=2) of p53 expression and ERK phosphorylation in the presence of DNA damage after p53 depletion revealing that silencing of p53 does not alter ERK expression and phosphorylation.
- **(B)** Analysis of ERK phosphorylation shows that p53 depletion does not affect the phosphorylation dynamics of ERK after DNA damage
- **(C)** Expression levels of p53 post siRNA mediated depletion in the presence of DNA damage.

(D) Expression of ERK and DNA damage response proteins ATM, CHK2 and p53 after DNA damage in the presence or absence of Ku-55933.

(E) Analysis of ERK phosphorylation and,

(F) p53 expression (F) shows that ATM inhibition (ATMi) with Ku-55933 results in the abrogation of p53 response and a significant attenuation of ERK phosphorylation (n=2).

(G) Fluorescence images of the stable MCF7-EKAREV cell lines expressing a FRET-based ERK activity reporter and a nuclear marker (mCherry-H2B) scale bar: 50 µm.

(H) Bootstrapped analysis of MCF7-EKAREV cells, serum starved, and treated with 130 ng/ml EGF.

(I) Bootstrapped analysis of MCF7-EKAREV cells, serum starved, and treated with 100 ng/ml HRG.

(J) Single cell traces used to compute the bootstrapped distributions shown in Fig 2C.

(K) Bootstrapped average and 95% confidence interval for the FRET ratios shown in Fig. 2 presented without normalization to control average. Normalization was necessary to detrend curves as we detect a decrease of apparent FRET during the first two hours of measurements.

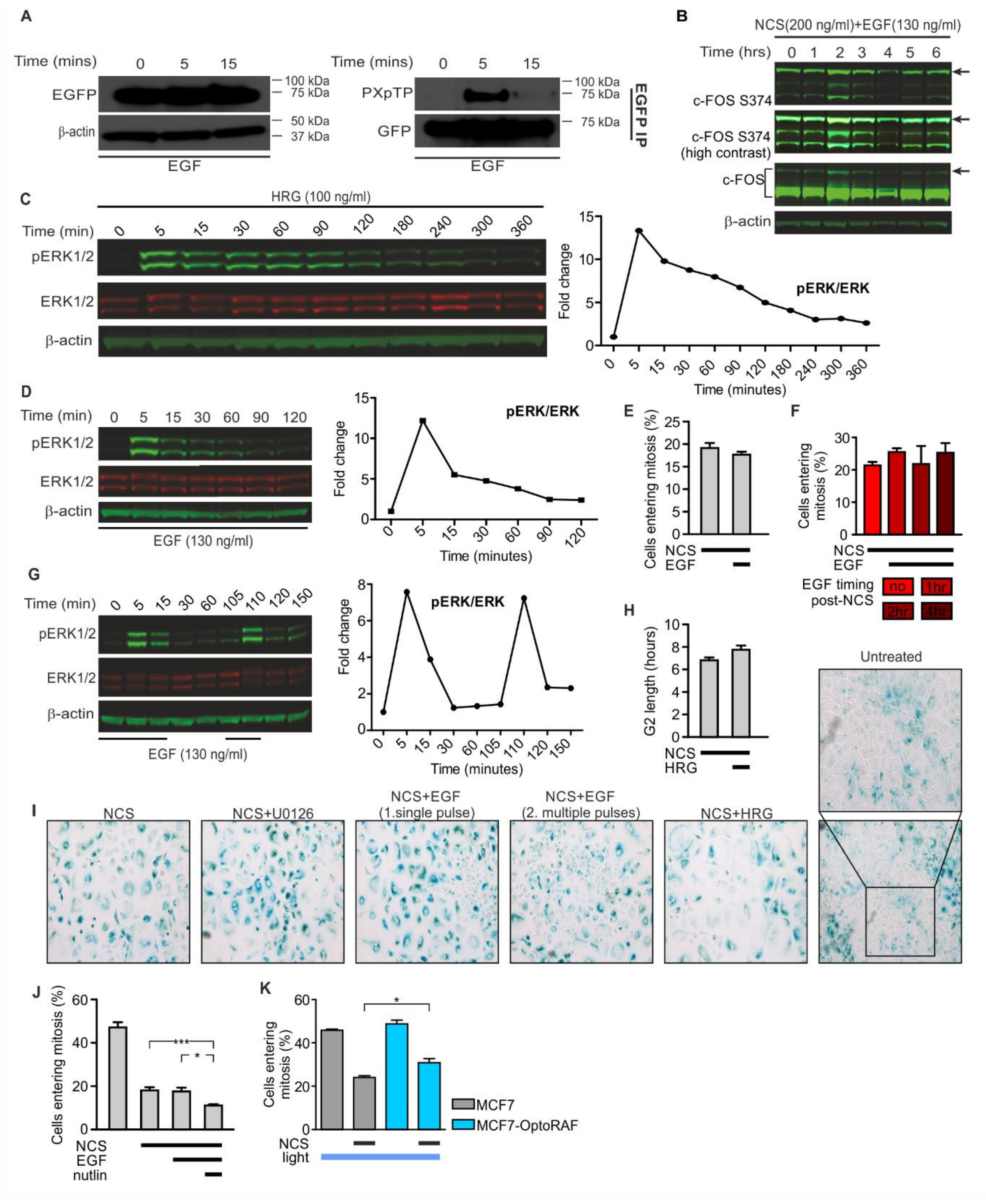

Figure S4. Validation of the MCF7-EKAREV cell line and growth factor treatments. Related to Figure 2.

- **(A)** Western blot analysis of MCF7-EKAREV cells treated with 100 ng/ml EGF for 5 and 15 minutes. The sensor was immunoprecipitated with a GFP antibody and the phosphorylation of the CDC25C T48 motif used in the sensor was confirmed by probing with anti-PXpTP antibody, which recognises phosphorylation only in the context of a phosphorylated Threonine followed by a Proline.
- **(B)** c-FOS phosphorylation after DNA damage and in the presence of EGF (HRG response shonw in Fig. 2, n=2). Arrows indicate c-FOS phosphorylated at Ser374.
- **(C)** Response of MCF7 cells to 100 ng/ml HRG induces sustained phosphorylation of ERK for several hours.
- **(D)** MCF7 cells treated with 130 ng/ml EGF exhibit a more transient response compared to HRG.

(E) Quantification of number of cells entering mitosis when treated with NCS and EGF shows no significant change between the to treatments thereby exhibiting that transient stimulation of MAPK pathway is not sufficient for allowing cells to move into mitosis in the presence of DNA damage (n=3).

(F) Pulses of MAPK activation induced by EGF treatment at different times do not change the fraction of cells released from G2 arrest (n=3).

(G) The transient nature of EGF response permitted us to use EGF to impose distinct pulses of MAPK activity with 130 ng/ml EGF for 15 minutes at the beginning of the experiments or after 105 minutes. Panel (G) shows two pulses separated by approximately two hours. Experiments were carried out in complete medium and cells were washed three times with complete medium 15 minutes after every EGF treatments.

(H) Quantification of time spent by MCF7 cells in G2 when treated with NCS and HRG shows no significant delay when compared to cells treated with NCS alone (n=3).

(I) Representative image of Beta gal staining of control and NCS-treated MCF7 cells in the presence of the MAPK inhibitor U0126, a single or multiple pulses of EGF and HRG, 4 days after treatment (related to Fig 2F)

(J) Quantification of number of MCF7 cells moving past G2 checkpoint and into mitosis when treated with NCS and EGF in the presence of Nutlin shows a stronger checkpoint arrest as compared to cells treated with NCS and NCS along with EGF (n=3).

(K) Contrary to transient stimulation, the sustained activation of MAPK signalling achieved with CRY2-cRAF increases the number of cells entering mitosis after DNA damage (NCS 200 ng/ml).

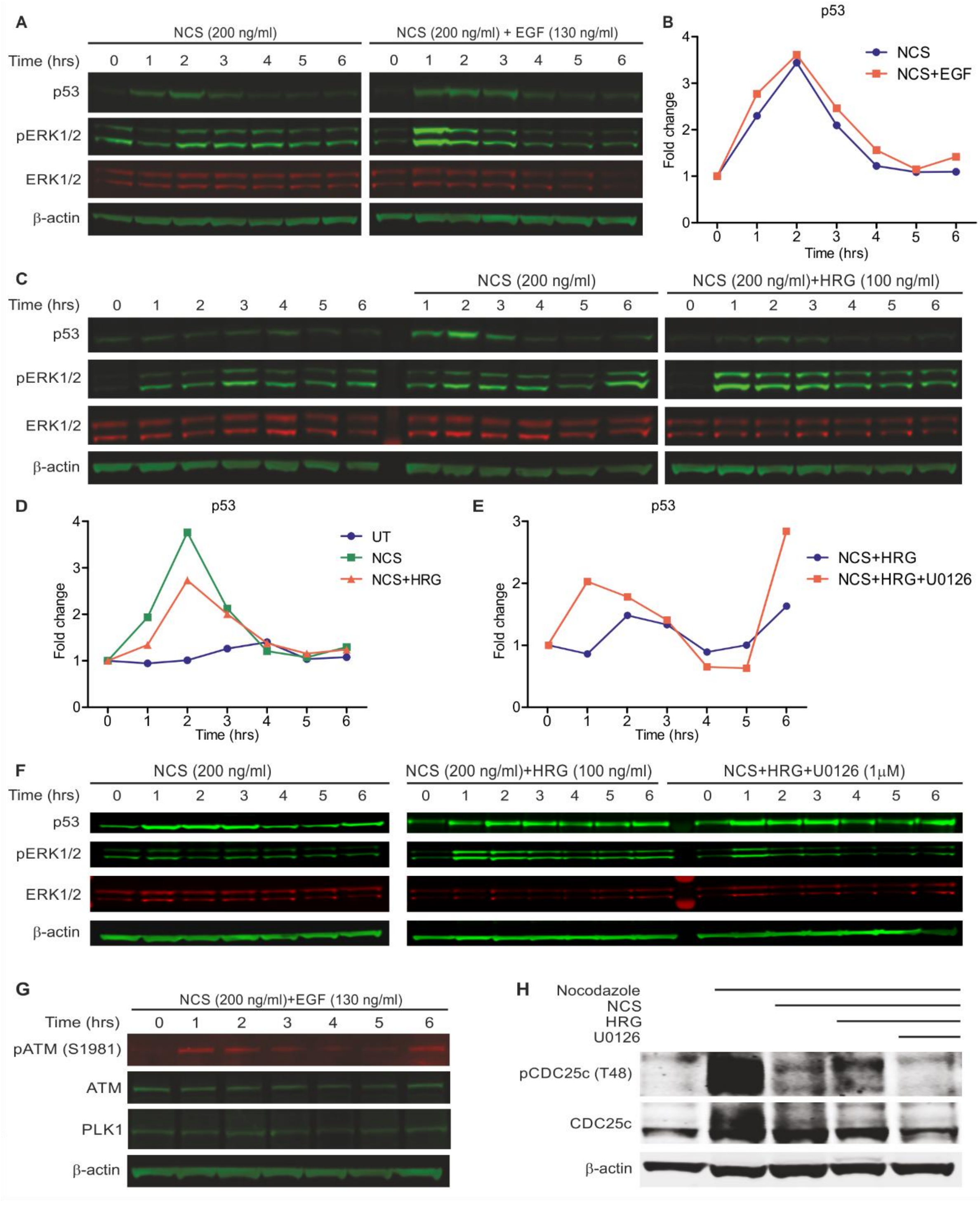

Figure S5. Sustained MAPK signalling attenuate p53 response and cell cycle arrest. Related to Figures 4.

- **(A)** Representative Western blot showing asynchronous MCF-7 cells treated with NCS in the absence or presence of EGF (130ng/ml, n=2).
- **(B)** Quantification of p53 expression as seen in panel A.
- **(C)** Representative Western blot showing MCF-7 cells synchronised by means of double thymidine block and released into fresh medium for 6 hrs before treatment with NCS or NCS and HRG.
- **(D)** Quantification of p53 expression levels as seen in panel C showing that HRG can attenuate the p53 response to DNA damage (n=3).
- **(E)** Analysis of p53 expression levels in cells treated with HRG and DNA damage in the presence and absence of U0126 shows that p53 levels can be rescued in the presence of U0126.

(F) Representative western blot showing MCF-7 cells treated with HRGand DNA damage in the absence/presence of U0126 (n=2).

(G) Contrary to HRG, an EGF pulse is unable to induce PLK1 expression in the presence of DNA damage as shown by a representative Western blot depicting PLK1 expression in MCF-7 cells treated with NCS (200 ng/ml) and EGF (130 ng/ml) (n=2).

(H) Representative western blot showing MCF-7 cells synchronised by a double thymidine block and subsequently released into fresh medium for 6 hours before addition of Nocodazole. At the same time, cells were treated with NCS alone, or in the presence of HRG or HRG+U0126. After 6 hr of additional incubations, cells were lysed and probed for CDC25C T48 phosphorylation and total CDC25C levels (n=3) showing that HRG can increase CDC25C phosphorylation in the presence of DNA damage in a MAPK-dependent manner.

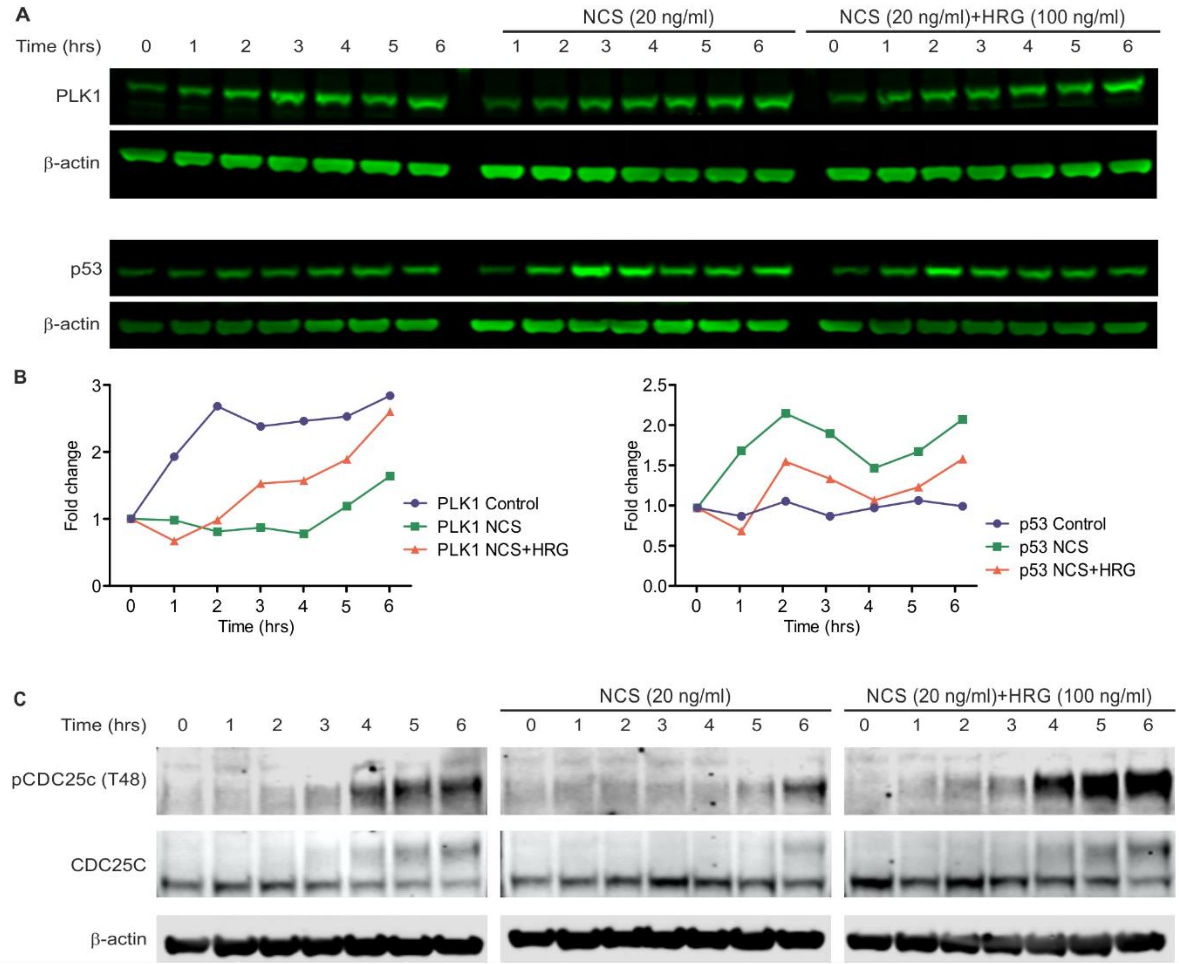

Figure S6. Sustained MAPK signalling attenuate p53 response and cell cycle arrest in RPE-1 cells. Related to Figure 4.

- **(A)** Representative Western blot showing RPE-1 cells synchronised by means of double thymidine block and released into fresh medium for 6 hrs before treatment with NCS or NCS and HRG.
- **(B)** Quantification of PLK1 expression levels as seen in panel A showing that HRG can increase the PLK1 even in presence of DNA damage (n=2). Analysis of p53 expression levels in cells treated with HRG and DNA damage in the shows that HRG can decrease the levels of p53.
- **(C)** Representative Western blot showing that CDC25C phosphorylation at T48 in synchronised RPE-1 cells is decreased in response to DNA damage and is restored by HRG (n=2).

dataset	p value	test
Fig. S3b – FOS	0.0085	ANOVA
Fig. S3b – EGR1	0.010	ANOVA
Fig. S3b – CTGF	0.0051	ANOVA
Fig. 1b	<0.0001	ANOVA
Fig. 1b	0.0059	ANOVA
Fig. 2e	0.0074	t test
Fig. 2f	<0.0001	ANOVA
Fig. 2g	<0.0001	ANOVA
Fig. 2j	<0.0001	ANOVA
Fig. 3e	<0.0001	ANOVA
Fig. S2c	<0.0001	ANOVA
Fig. S2e	<0.0001	ANOVA
Fig. S5j	<0.0001	ANOVA
Fig. S5k	<0.0001	ANOVA

Supplementary Table. Related to Fig. 1, 2, 3, S2, S4. Exact p values (unless <0.0001) and statistical tests. ANOVA: one-way ANOVA with Newman-Keuls multiple comparison test; t-test: two tailed paired t-test
